# Searching for novel MDM2/MDMX dual inhibitors through a drug repurposing approach

**DOI:** 10.1080/14756366.2023.2288810

**Published:** 2023-12-07

**Authors:** Keting Li, Wenshu Hu, Yingjie Wang, Wenxing Chen, Hongmei Wen, Jian Liu, Wei Li, Bo Wang

**Affiliations:** School of Pharmacy, Nanjing University of Chinese Medicine, Nanjing, China

**Keywords:** Drug repurposing, MDM2/MDMX, p53, nintedanib, dual inhibitors

## Abstract

Disruption of p53-MDM2/MDMX interaction by smaller inhibitors is a promising therapeutic intervention gaining tremendous interest. However, no MDM2/MDMX inhibitors have been marketed so far. Drug repurposing is a validated, practical approach to drug discovery. In this regard, we employed structure-based virtual screening in a reservoir of marketed drugs and identified nintedanib as a new MDM2/MDMX dual inhibitor. The computational structure analysis and biochemical experiments uncover that nintedanib binds MDM2/MDMX similarly to RO2443, a dual MDM2/MDMX inhibitor. Furthermore, the mechanistic study reveals that nintedanib disrupts the physical interaction of p53-MDM2/MDMX, enabling the transcriptional activation of p53 and the subsequent cell cycle arrest and growth inhibition in p53^+/+^ cancer cells. Lastly, structural minimisation of nintedanib yields H3 with the equivalent potency. In summary, this work provides a solid foundation for reshaping nintedanib as a valuable lead compound for the further design of MDM2/MDMX dual inhibitors.

## Introduction

p53, A pivotal tumour suppressor, plays critical roles in cell cycle regulation, apoptosis, and DNA repair^1–3^, by itself or by inducing the expression of downstream targets from internal or external cellular stress signals^4^. *TP53* mutations have been found in approximately half of the human cancers, while wild-type p53 in the rest of the malignancies are often functionally inactivated because of the overexpressed negative regulators mouse double minute 2 (MDM2) and mouse double minute X (MDMX)^5^. Upon binding, MDM2 and MDMX squelch the function of p53 cooperatively^6^, either by blocking the DNA binding site or inducing the proteasomal degradation of p53. Most MDM2 inhibitors show weak interaction with MDMX, thus compromising the pharmacological effect in MDMX overexpressed cells^7^; therefore, dual blockade of p53-MDM2/MDMX interaction is a promising therapeutic strategy.

In our previous work of discovery of MDM2/MDMX dual inhibitor (**C16**)^8^^,^^9^, structural superimposition demonstrated that the two proteins share similar binding sequences, with an ααβαβα conformation. Moreover, molecular mechanics with generalised Born and surface area solvation (MM/GBSA) calculation and alanine scanning were employed, confirming the hot-spot residues of MDM2 (Val14, Leu54, Ile61, His96, and Ile99) and MDMX (Met53, Leu56, Ile60, and Leu98). All these results provide the foundation for discovering MDM2/MDMX dual inhibitors.

**Figure 1.**
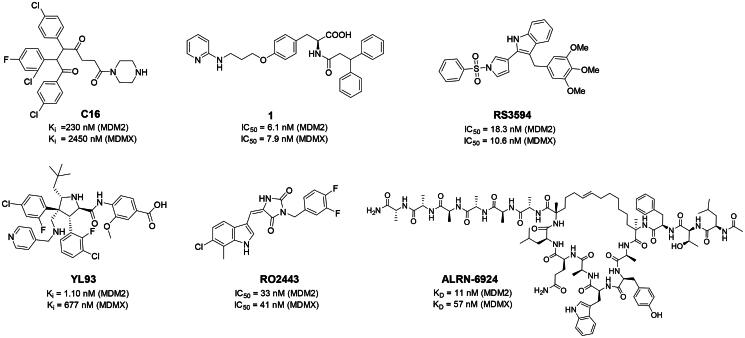
The representative MDM2/MDMX dual inhibitors.

Numerous MDM2/MDMX dual inhibitors have been developed and reported (Figure 1)^10^. Marinelli and colleagues reported compound **1** and **RS3594** by virtual screening of an in-house database. Compound 1 demonstrates single-digit nanomolar affinity to MDM2/MDMX, and initiates inhibition of cell proliferation with an IC_50_ of 356 nM in neuroblastoma SHSY5Y cells^11^. RS3594 possesses strong binding affinity to both targets and exerts cell growth inhibition synergically with CXCR4 antagonist in glioblastoma cells. Recently, **YL93** was discovered based on the structure of MDM2 inhibitor idasanutlin^12^. YL93 is a potent MDM2/MDMX dual inhibitor with higher cellular potency. The appended ethylpyridyl moiety forms a crucial H-bond with His55 of MDM4. **RO2443** is a potent MDM2/MDMX dual inhibitor with IC_50_ of 33 nM and 41 nM for MDM2 and MDMX, although with poor solubility and cellular efficacy. Nevertheless, RO2443 binds MDM2 and MDMX in a 2 + 2 sandwich mode^13^. This unique feature inspires our work in this paper (*vide infra*). Instead of smaller molecular inhibitors, Sulanemadlin (**ALRN-6924**), a cell-penetrating staple peptide, exerts dual inhibition on MDM2 and MDMX with nanomolar range affinity^14^. The phase I trial has been completed, showing the well tolerance of sulanemadlin. It displays strong antitumor efficacy in solid tumours and lymphoma harnessing wild-type p53^15^, which is now in phase II clinical trial (NCT03725436). Despite the recent progress, simultaneous inhibition of MDM2/MDMX with drug-like molecules is still challenging.

Instead of the traditional drug discovery process, drug repurposing is a tangible, validated tactic with salient characteristics, such as shorter development time, lower cost, and better safety profile. It employs existing drugs or drug candidates for novel indications or targets^16^. In this work, we applied drug repurposing to discover novel MDM2/MDMX dual inhibitors and virtually screened a reservoir of FDA-approved drugs. Among them, nintedanib^17^^,^^18^, an FDA-approved multikinase inhibitor for Idiopathic Pulmonary Fibrosis (IPF), showcased the potential as a new MDM2/MDMX dual inhibitor, and this was validated by a series of structural and biochemical analyses. Nintedanib forms a strong bind with MDM2 (*K*_d_ = 1.79 ± 0.34 μM) and MDMX (*K*_d_ = 0.72 ± 0.40 μM), respectively. Moreover, it exerts a potent antiproliferative effect in MDM2/MDMX overexpressed cancerous cells. Lastly, structural minimisation of nintedanib was performed, and the preliminary structure-activity relationship (SAR) was revealed, yielding H3 with the equivalent potency. Taken together, our work lays the foundation for repurposing nintedanib as a lead compound for further development of MDM2/MDMX dual inhibitors.

## Results and discussion

### Virtual screening of FDA-approved drugs for potential MDM2/MDMX dual inhibitors

**Figure 2.**
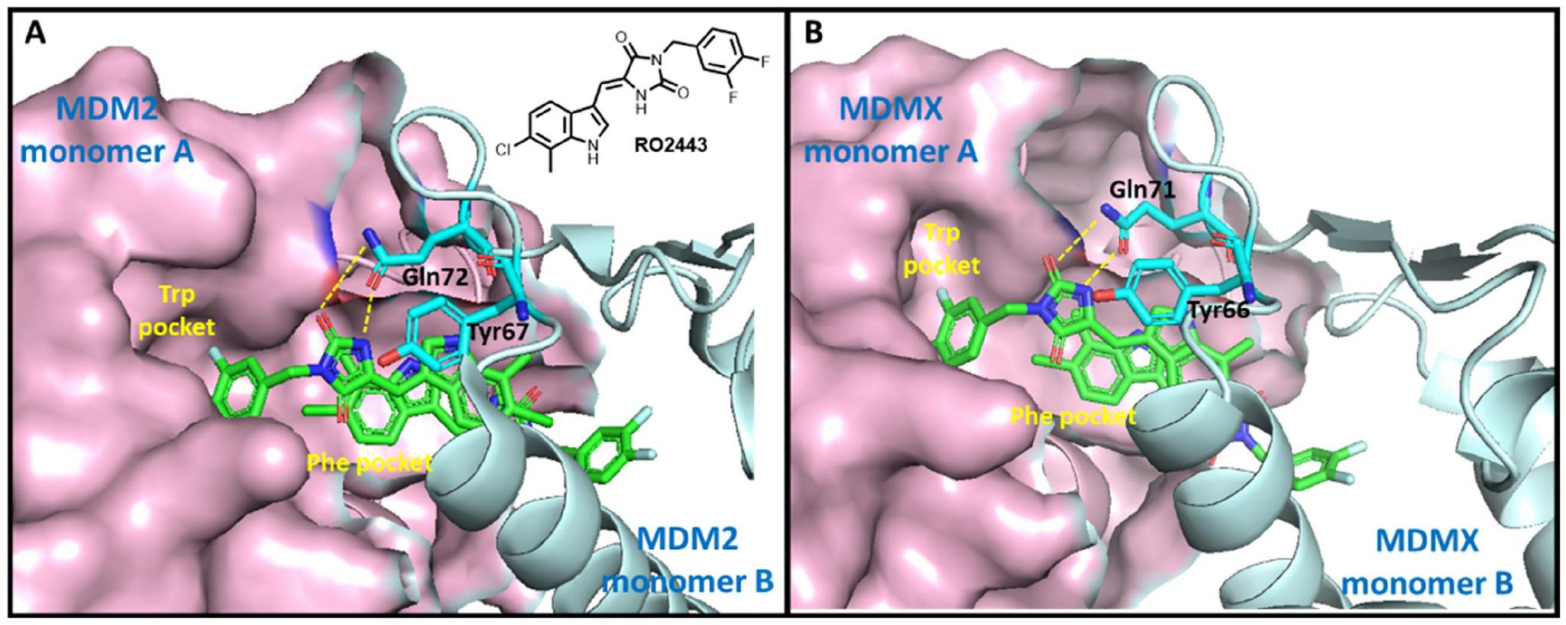
The binding modes of RO2443 with MDM2 and MDMX. One protein monomer is shown as a light-pink surface, and the other as an aquamarine cartoon. (A) The co-crystal structure of RO2443 with MDM2 (PDB: 3VBG); (B) The co-crystal structure of RO2443 with MDMX (PDB: 3U15).

MDM2 and MDMX are homologs and share a similar p53 binding domain (similar peptide sequence and 3D structure). The interaction of p53-MDM2/MDMX is mediated by hydrophobic interactions, specifically, the interactions of Phe19, Trp23, and Leu26 of p53 with multiple conserved MDM2/MDMX hydrophobic residues^19^^,^^20^. As abovementioned, RO2443 is a potent dual inhibitor of MDM2 and MDMX^13^. RO2443 binds MDM2 or MDMX in a ratio of 2:2 to form a homodimer. As shown in Figure 2, when bound, the difluorophenyl ring of RO2443 inserts into the Trp pocket of protein monomer A, whereas the indolyl-hydantoin moiety occupies the Phe pocket of protein monomer B. A four-level π-π stacking interaction forms across the two indolyl-hydantoin and two Tyr67 residues (Tyr66 in the case of MDMX)^13^. H-bonds are also observed between carbonyl, NH of hydantoin, and acetamide of Gln72 of MDM2 (Gln71 in MDMX). Our molecular redocking study also confirmed the similar binding modes and interactions (Figure S1 and S2). Owing to the detailed structural information, we chose the crystallographical structures of RO2443-MDM2/MDMX as models to carry out the following virtual screening.

**Figure 3.**
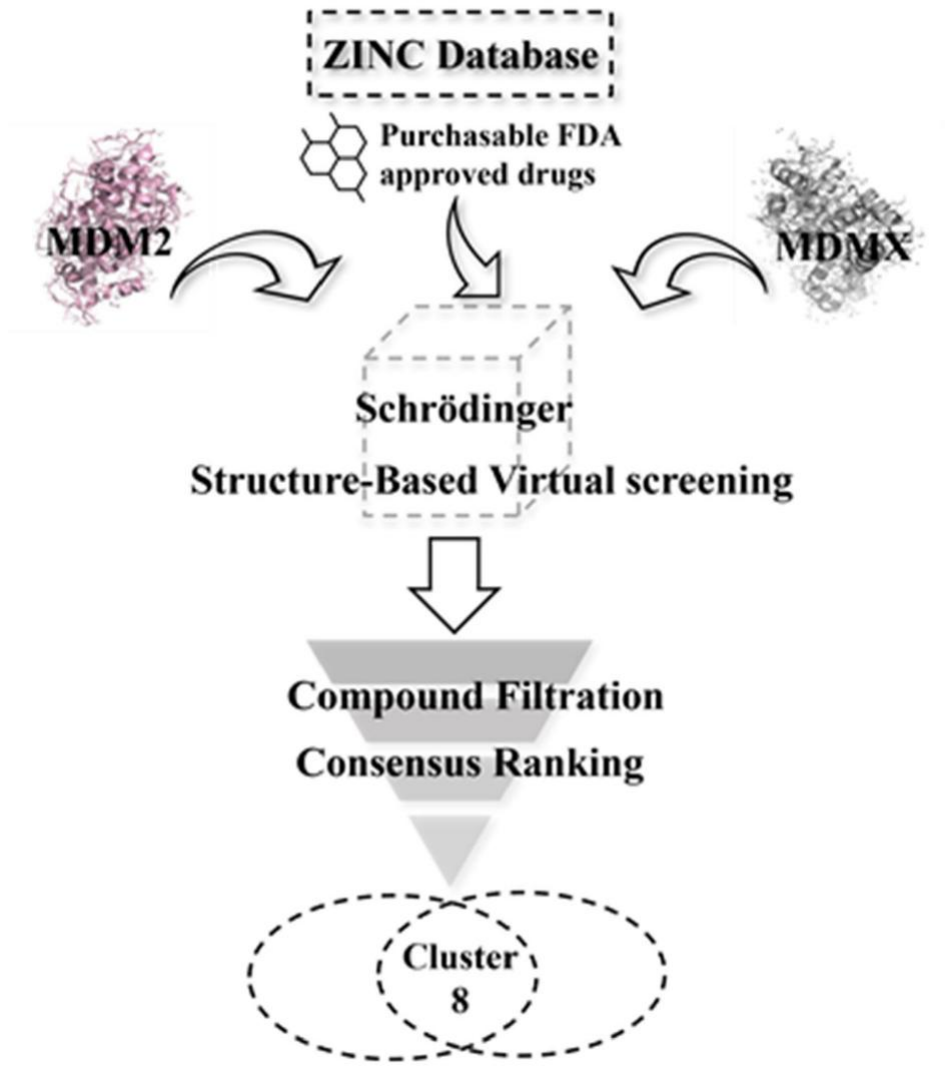
Virtual screening workflow for the identification of MDM2/MDMX dual inhibitors.

The workflow of virtual screening is illustrated in Figure 3. First, 1355 purchasable FDA-approved drugs in the ZINC database were docked into the binding sites of MDM2 and MDMX using Schrödinger 2019, which generated docking poses and scores for these compounds. We analysed the interaction between the compounds and binding sites to enhance the hit rate. The compounds that do not form hydrophobic interactions with the surrounding residues were filtered out. 55 and 60 drugs were identified for MDM2 and MDMX, respectively. Although these compounds are FDA-approved drugs, they were re-screened by Lipinski and Veber’s rules to ensure they meet these criteria. Hydrogen bond is another vital force for the drug-target interaction. Then, the docking structures were scrutinised and compounds forming hydrogen bonds with both proteins were picked out. Finally, the resulting 8 compounds (Table 1) were tabulated and ranked based on the docking scores. Among them, nintedanib was picked for further investigation due to its higher docking score and similar binding mode to RO2443 (Figure 3). Nintedanib is an FDA-approved triple angiokinase inhibitor for the treatment of Idiopathic Pulmonary Fibrosis, which works by targeting vascular endothelial growth factor receptor (VEGFR), platelet-derived growth factor receptor (PDGFR), and fibroblast growth factor receptor (FGFR)^18^. In addition, Nintedanib has also been recognised as an anticancer agent in various cancers, such as ovarian cancer^21^, hepatocellular carcinoma^22^, and renal cancer^23^. However, the exact mechanism of action (MOA) is still elusive.

**Table 1.** Docking scores of selected drugs against MDM2 and MDMX.

ZINC ID	Generic name	Structure	Docking Score	
			MDM2	MDMX
ZINC100014909	Nintedanib	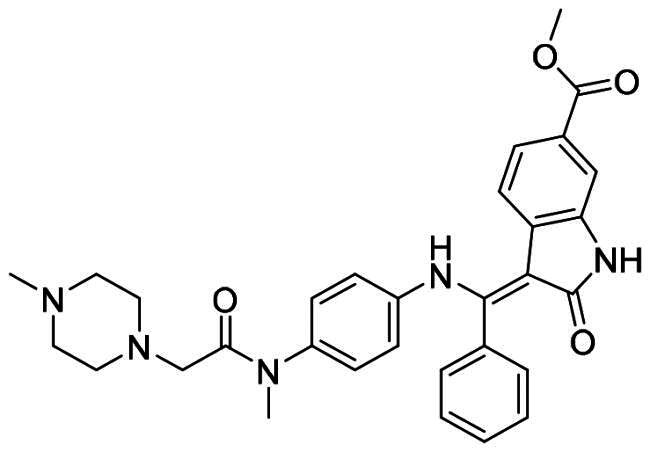	−7.905	−9.153
ZINC3810860	Zetia	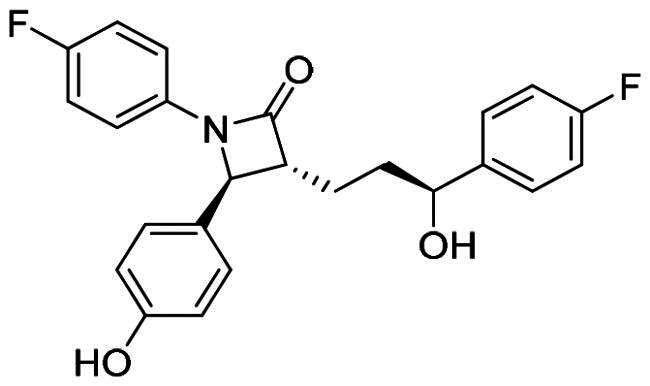	−8.054	−8.031
ZINC3874185	Mefloquine	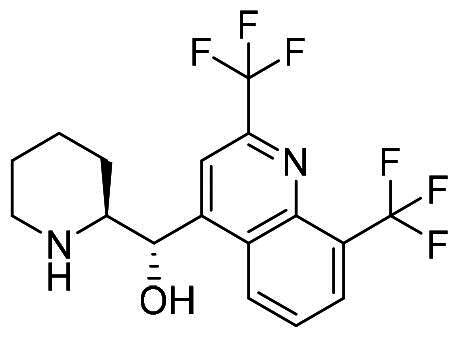	−8.157	−7.698
ZINC896595	Ativan	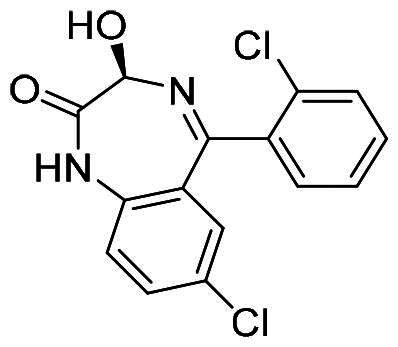	−7.782	−6.769
ZINC3831490	Azulfidine	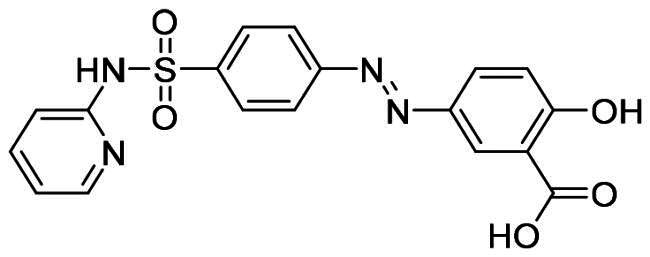	−6.131	−7.165
ZINC21303210	Brimonidine	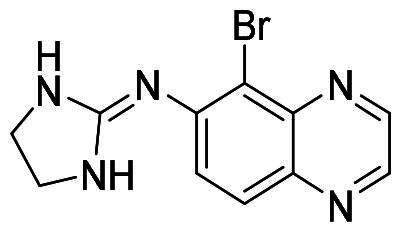	−6.007	−6.336
ZINC3952881	Balsalazide	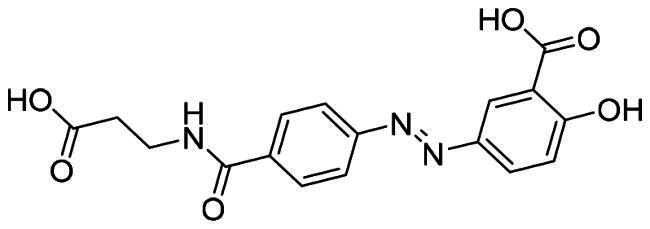	−5.194	−6.606
ZINC19702309	Tizanidine	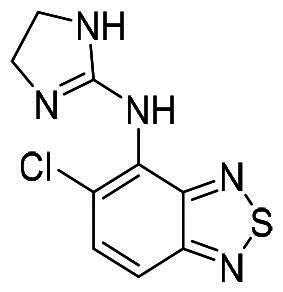	−5.316	−6.447

**Figure 4.**
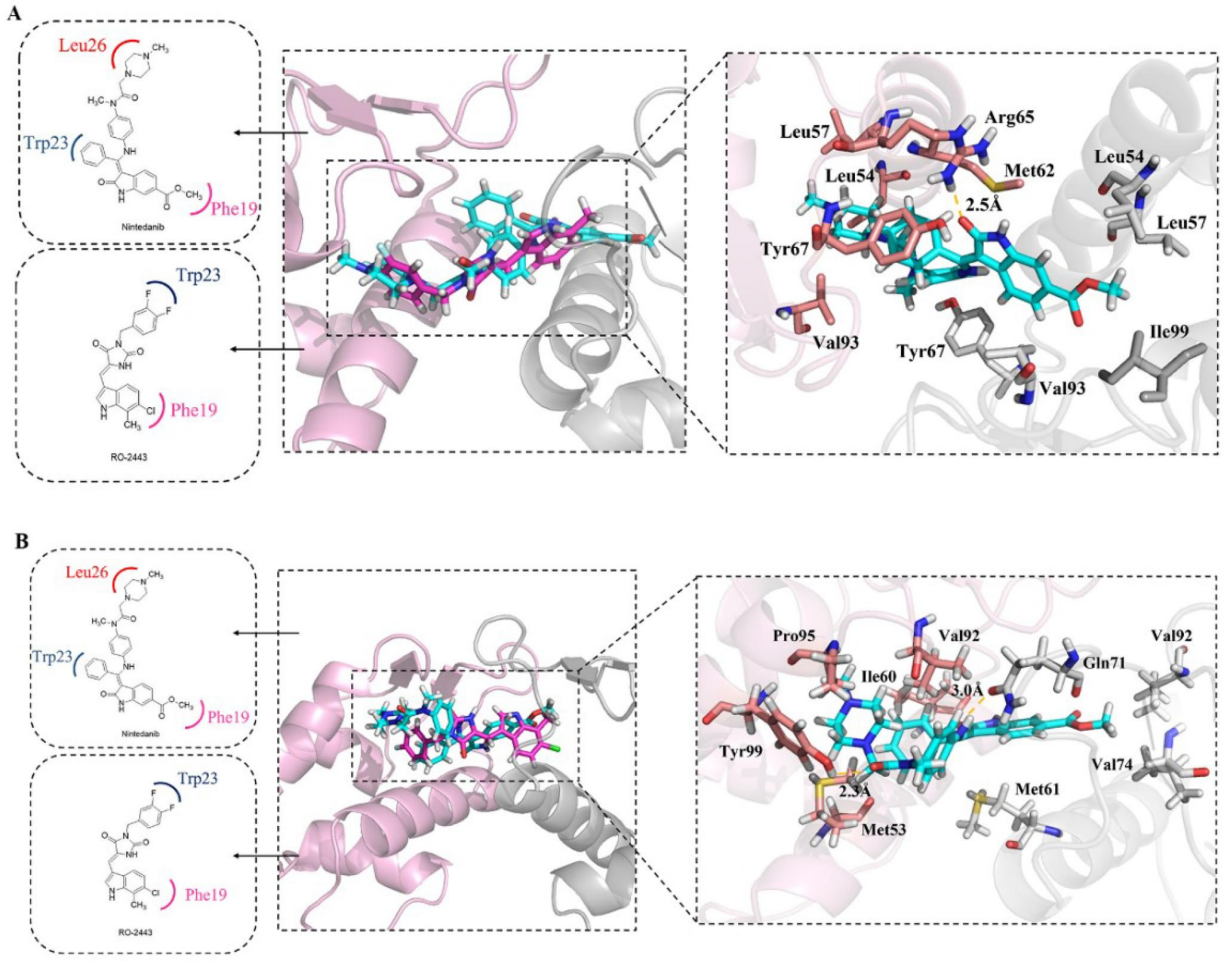
Molecular docking analysis of nintedanib with MDM2 and MDMX. For explicitly, another molecule of nintedanib was removed. In A and B, the pink ribbon represents one MDM2 or MDMX monomer, and the gray ribbon represents another. (A) The superimposition of nintedanib (cyan) and RO2443 (magenta) in MDM2 dimer. The docking mode of nintedanib with MDM2 (PDB ID: 3VBG) is represented as a ribbon with the critical amino acid side chain explicitly shown (right). (B) The superimposition of nintedanib (cyan) and RO-443 (magenta) in MDMX dimer. The docking mode of nintedanib with MDMX (PDB ID: 3U15) is represented as a ribbon with the critical amino acid side chain explicitly shown (right). The yellow dotted line represents the hydrogen bonds in (A) and (B).

The binding modes of nintedanib with MDM2 and MDMX were profiled in Figure 4. The dihydroindolyl moiety of nintedanib occupies the Phe pocket of one protein monomer, while the phenyl group inserts into the Trp pocket of another. In addition, the piperazine moiety of nintedanib stretches further into the Leu pocket. Owing to the similar structure feature of nintedanib with RO2443 and molecular docking, a ratio of 2:2 binding mode was speculated in the case of nintedanib-MDM2/MDMX complexes, which need to be confirmed by other techniques.

The more detailed interactions of nintedanib with MDM2 and MDMX were scrutinised. Nintedanib forms hydrophobic interactions with the critical amino acid residues of one monomer, such as Leu54, Leu 57, Met 62, Tyr67, Val93, and Leu54, Leu57, Tyr67, Val 93, Ile99 of another (Figure 4A). Similarly, the interaction of nintedanib-MDMX is mediated by Met61, Val74, Val92 of one monomer and Met53, Ile60, Val92, and Pro95 of another (Figure 4B). Among them, Leu54, Ile99 of MDM2, and MeT53, Ile60 of MDMX are the conserved hot-spot residues, which is in line with our previous study^8^^,^^9^. Furthermore, the oxygen of 2-oxoindoline of nintedanib forms an H-bond with Arg65 (2.5 Å) of MDM2. For MDMX, two H-bonds were observed, in which carbonyl oxygens of ester and amide form hydrogen bonds with Gln71 (3.0 Å) and Tyr99 (2.3 Å), respectively. Owing to the one more hydrogen bond in the nintedanib-MDMX complex, it might indicate a better binding affinity.

### Stability of nintedanib-MDM2/MDMX complexes during MD simulation

**Figure 5.**
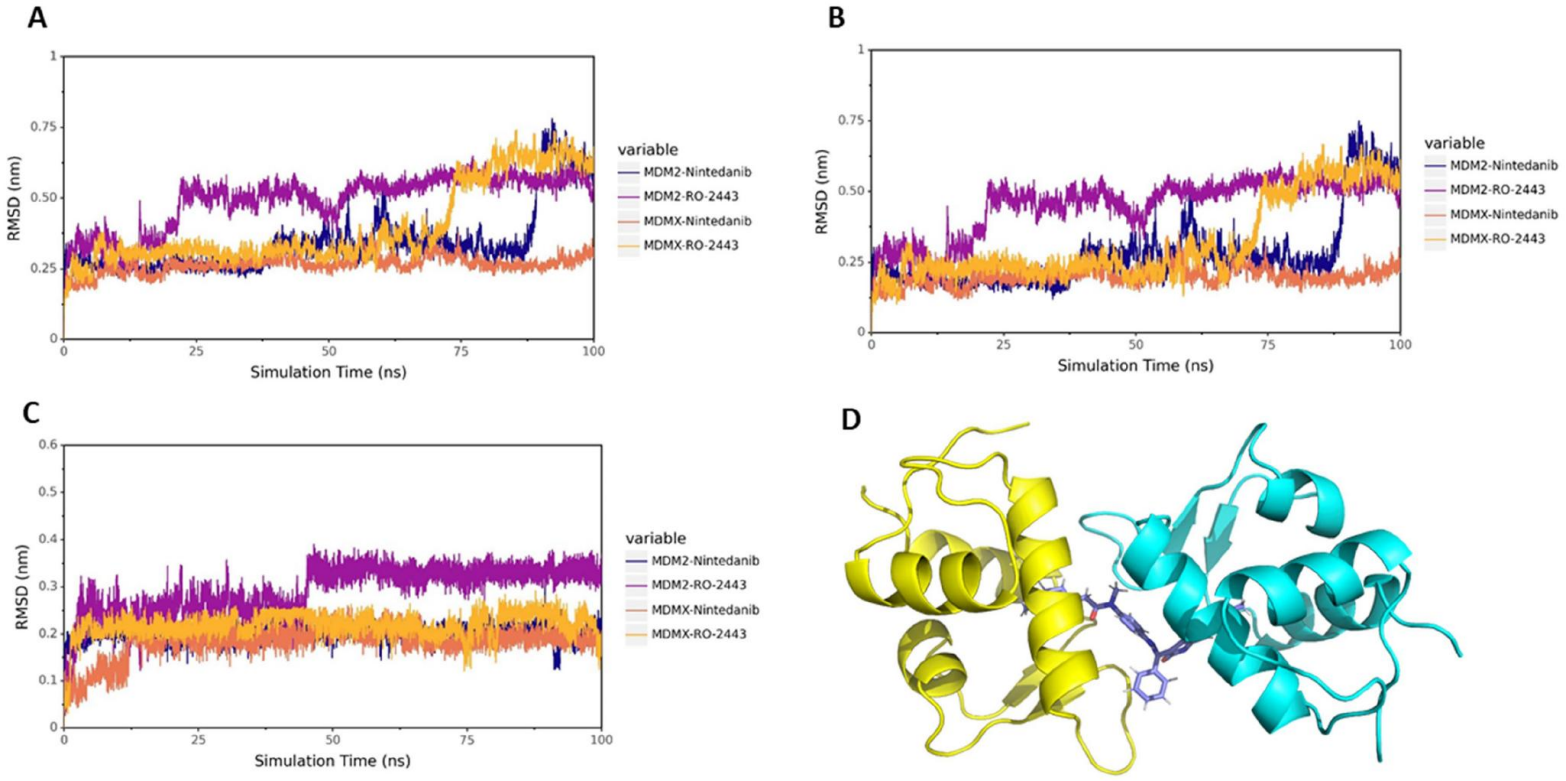
MD simulation analysis by GROMACS. (A) The protein backbone RMSD of proteins in complex with different ligands. (B) The protein backbone RMSD of proteins. (C) The protein backbone RMSD of ligands. (D) The binding mode of MDM2 with nintedanib (purple). For explicitly, one molecule of nintedanib was presented. The two chains of MDM2 were shown as yellow and cyan ribbon, respectively.

To obtain the stable binding conformation and validate the docking results, MD simulation (100 ns) was performed by GROMACS^24^. Initially, we assessed the structural stability by plotting the structural fluctuation of the protein backbone. Root mean square deviation (RMSD) was assigned to profile the protein backbone difference between the initial and final conformation. The fluctuation is dramatic, as well as the complex movement. As shown in Figure 5, the RMSD of the complexes MDM2-nintedanib and MDMX-RO2443 showed more drastic fluctuation. However, in the non-fluctuating period, the RMSD remained stable. To study the origin of the instability of the complex, we plotted the RMSD of proteins and ligands separately (Figure 5B,C). The results indicated that the fluctuations were coming from the protein part. As shown in Figure 5(D), the MDM2 protein consists of two chains, which are not intertwined but bind together through the interface. So, the structure is not tight and is prone to change, leading to fluctuations.

The hydrogen bond is one of the most vital non-covalent interactions in drug-target binding. Therefore, we performed the H-bond occupancy analysis along with the elongation of the simulation time, as shown in Figure S6. The left panel shows the donor, acceptor and occupancy of hydrogen bonding; the right panel shows the corresponding hydrogen bond formation frequency during 100 ns. The denser the bar line is, the higher the frequency of hydrogen bond forms. The H-bond formation analysis aligns with the molecular docking results. In particular, the H-bond between Arg65 of MDM2 with nintedanib disappeared, and the new H-bond was forged with Tyr67 at 100 ns (Figure S3 and S6A). A similar phenomenon was also observed in the case of MDMX with nintedanib at 100 ns, where an H-bond was possibly formed between Gln58 of MDMX.

### Binding free energy analysis of nintedanib-MDM2/MDMX complexes

In the previous section, we discussed the stability of nintedanib-MDM2 and MDMX complexes. We then set out to calculate the binding free energy of the complexes using the end-point MM/GBSA method^25^ based on the snapshots of the complexes sampled from their respective MD trajectories. Nintedanib exhibits strong binding affinities with both proteins (Table 2). As shown below, the Van der Waals force plays the most significant role in the binding process. Notably, the van der Waals forces in the protein-nintedanib complexes are all greater than those of RO2443. In addition, the binding energy of the nintedanib-MDMX complex is lower than that of the nintedanib-MDM2 complex, indicating a tighter binding with MDMX. By decomposing the binding energy ΔG_bind_, the contribution of each amino acid to the total binding energy can be obtained. The residues that contribute more to the binding energy in each group were shown in Figure S1–S4, which is in accordance with the previous study and molecular docking.

**Table 2.** Binding free energies and energy components predicted by MM/GBSA (kcal/mol).

System name	MDMX-nintedanib	MDM2-nintedanib	MDMX- RO2433	MDM2- RO2433
ΔE_vdw_	−287.302 ± 2.015	−244.556 ± 16.729	−155.209 ± 1.751	−224.941 ± 1.11
ΔE_elec_	−30.548 ± 0.932	−27.287 ± 5.893	−14.306 ± 1.247	−15.887 ± 0.752
ΔE_pol_	174.897 ± 1.867	156.321 ± 22.715	99.987 ± 2.861	138.091 ± 0.968
ΔE_nonpol_	−33.74 ± 0.04	−32.509 ± 1.039	−21.663 ± 0.213	−22.299 ± 0.054
ΔG_bind_	−176.693 ± 2.46	−148.03 ± 16.909	−91.19 ± 4.904	−125.037 ± 1.751

ΔE_vdw_: van der Waals energy; ΔE_elec_: electrostatic energy; ΔE_pol_: polar solvation energy; ΔE_nonpol_: non-polar solvation energy; ΔG_bind_: binding free energy.

### Nintedanib binds MDM2 and MDMX with high affinities

**Figure 6.**
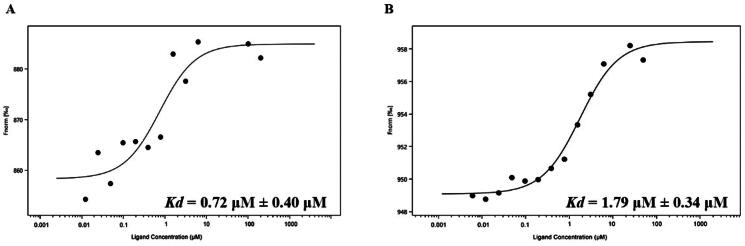
The binding affinities of nintedanib to MDM2 and MDMX. (A) The fitting curve of nintedanib to MDMX (1–110) in MST assay. (B) The fitting curve of nintedanib to MDM2 (1–110) in MST assay.

To further validate and characterise the interactions between nintedanib and MDM2/MDMX, a microscale thermophoresis (MST) experiment was performed using recombinant MDM2 (residues 1–110) and MDMX (residues 1–110) protein. The dissociation constant (*K*_d_) of nintedanib is 0.72 ± 0.40 μM for MDMX (Figure 6A) and 1.79 ± 0.34 μM for MDM2 (Figure 6B), indicating a higher affinity to MDMX. These results are correlated with the molecular docking and MD simulation. Due to the validated binding to MDM2 and MDMX, nintedanib could be a lead compound for developing MDM2/MDMX dual inhibitors.

### Nintedanib inhibits the proliferation and migration of cancer cells with wild-type p53

**Figure 7.**
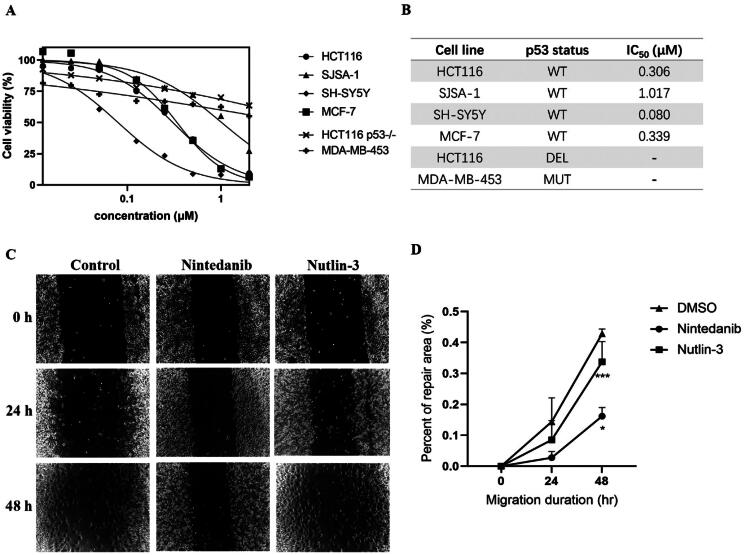
Antiproliferation of nintedanib against tumour cells. (A) Antiproliferative effect of nintedanib in cancer cells. Cells with wild-type p53 (HCT116, SJSA-1, SH-SY5Y, and MCF-7), mutant p53 (MDA-MB-453), or deleted p53 (HCT116) were incubated with indicated concentrations of nintedanib for 48 h, and the CCK-8 assay measured cell viability. (B) IC_50_ values of nintedanib in different cell lines (WT: wild type; DEL: deleted; MUT: mutant). (C and D) The effect of nintedanib on the SH-SY5Y cells migration. The data were presented as the means ± SD of three independent experiments. The significance of the differences was determined using one-way ANOVA with Bonferroni post-test. (* represents significant difference,*p < 0.05, ***p < 0.001).

As a canonical MDM2 inhibitor, it can disrupt MDM2-p53 interaction and reactivate the tumour suppression of p53, thus inducing cell growth inhibition^9^^,^^26^. Accordingly, we set forth to evaluate the *in vitro* antiproliferative activity of nintedanib in four human cancer cell lines with wild-type p53, namely HCT116, SJSA-1, MCF-7, and SH-SY5Y, along with p53-deleted HCT116 and p53-mutant MDA-MB-453 cells. As shown in Figures 7(A,B), nintedanib exhibits good cell growth inhibition in different tumour cells, with IC_50_ values of 0.306, 1.017, 0.339, and 0.080 μM in HCT116, SJSA-1, MCF-7, and SH-SY5Y cells, respectively. The antiproliferative activities against HCT116 (p53 deleted) and MDA-MB-453 (mutant p53) were also tested to examine the selectivity further. Nintedanib shows apparent growth inhibition in neither of the cells (Figure 7A,B). In brief, nintedanib inhibits cancerous cell proliferation in a p53-dependent manner, which behaves like a bona fide MDM2/MDMX dual inhibitor.

Furthermore, the wound-healing assay was performed to evaluate the cell migration of SH-SY5Y cells upon treatment with nintedanib. The repair rate for the nintedanib-treated group is lower than the control and nutlin-3-treated groups (Figures 7C,D), demonstrating that nintedanib can effectively prevent cancer cell migration.

### Nintedanib activates the transcriptional function of p53 in cancer cells with wild-type p53

**Figure 8.**
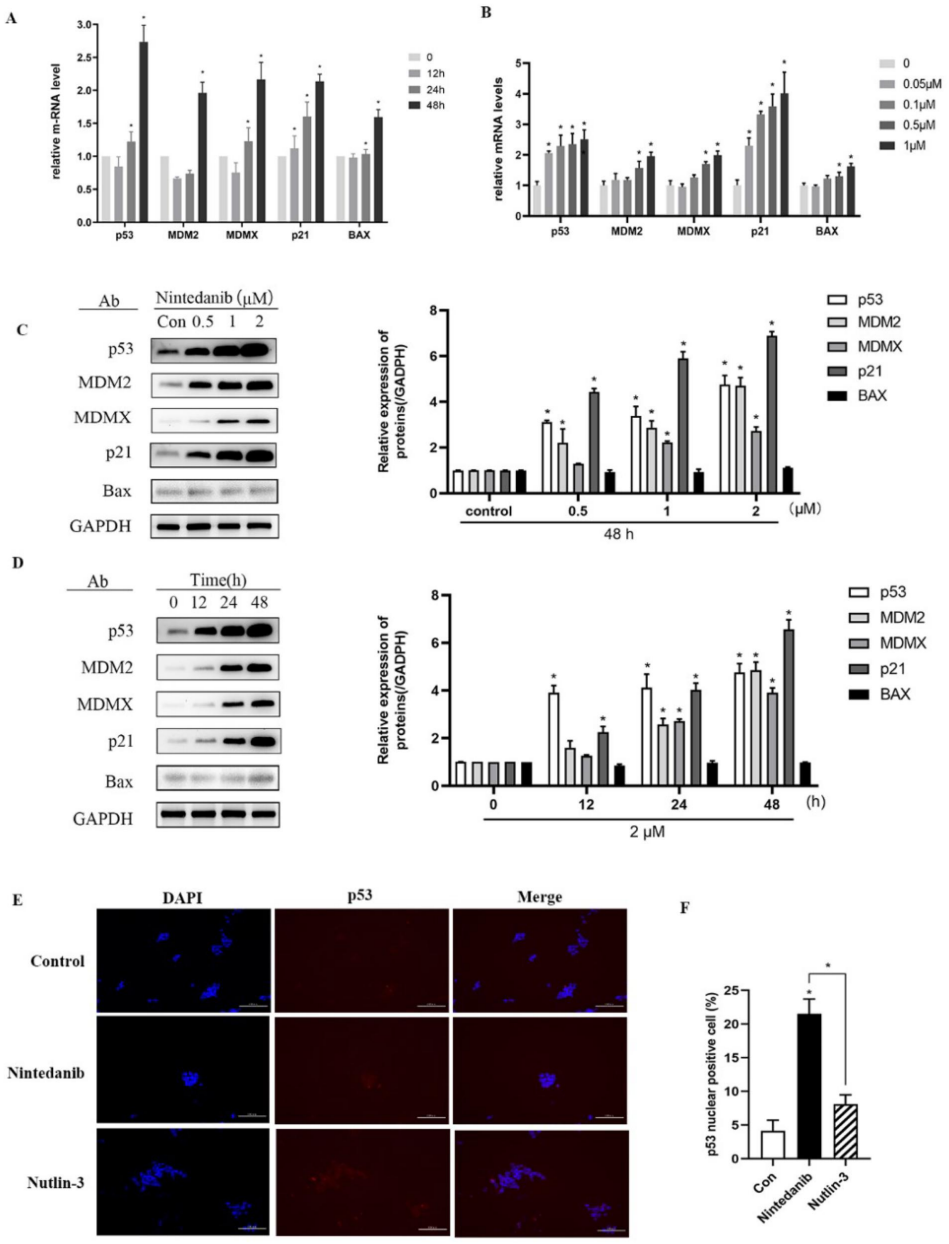
Nintedanib activates the p53 pathways in HCT116 cells. (A and B) RT-qPCR analysis of mRNA levels of p53 and targeted genes in HCT116 cells. Cells were treated with DMSO and indicated concentrations of nintedanib at different time courses. (C and D) Immunoblot of p53 and its downstream factors following treatment with DMSO and indicated concentrations of nintedanib at different time courses. GADPH was used as the loading control. Band intensities of blot C were calculated by Image J, and the bar graph was plotted and fitted by GraphPad Prism 8. (E and F) Immunofluorescence detection of p53. Red: Sulfo-Cyanine3 (CY3) staining shows the presence of p53 (red dots). Nuclei were counterstained with DAPI (blue). Scale bar =100 μm. Cells with p53 nuclear positive were quantified by counting 100 cells (magnification 200 ×) for each treatment. The data were presented as the mean ± SD of three experiments in all experiments. The result was normalised and statistically analysed in one-way ANOVA with Bonferroni post-test (*p < 0.05).

A comprehensive investigation was pursued to uncover the MOA underlying cell growth inhibition. Firstly, we investigated the mRNA levels of p53 and its main transcriptional targets MDM2, MDMX, p21, and BAX. Real-time PCR was conducted after treating HCT116 cells (p53^+/+^) with nintedanib for 12 h. The p53 mRNA level is increased in time-dependent and dose-dependent ways, as well as those of p53 targeted genes (Figures 8A,B). Furthermore, immunoblotting analysis was conducted to evaluate the effect of nintedanib on the protein levels (Figure 8C). Upon treatment, the p53 level is augmented dose-dependently, as well as the MDM2, MDMX, p21, and BAX levels. HCT116 cells were treated with 2 µM of nintedanib at different time courses (Figure 8D), demonstrating a time-dependent upregulation.

The transcriptional activity of p53 depends on its protein level and, more importantly, on its localisation in the nucleus. Therefore, an immunofluorescence assay was employed to visualise the nuclear translocation of p53. Compared to the control group, nintedanib induces more p53 accumulated in the nuclei of HCT116 cells, even more efficiently than the nutlin-3 treated group (Figures 8E,F). Taken together, all these results demonstrate that nintedanib could disrupt MDM2/MDMX-p53 interaction, thus reactivating the transcriptional function of p53 in a dose- and time-dependent manner.

### Nintedanib promotes the G2/M cell cycle arrest in cancer cells with wild-type p53

**Figure 9.**
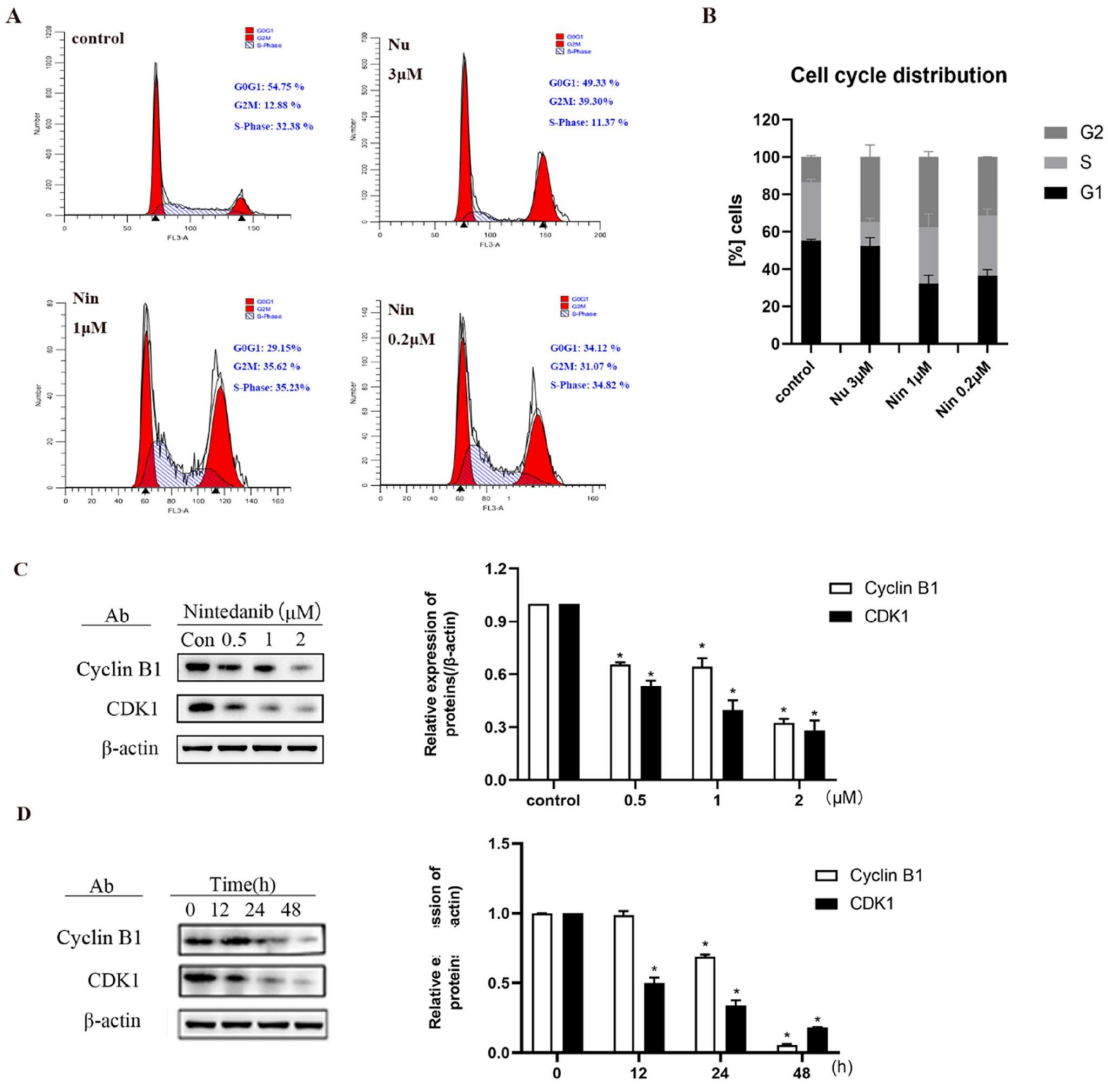
Nintedanib promotes cell cycle arrest in the G2/M phase of HCT116 cells. (A) Nintedanib induces cell cycle arrest in HCT116 cells. Cells were treated with DMSO, 1 or 0.2 μM of nintedanib, or 3 μM of nutlin-3 (positive control) for 48h. Cell cycle distribution was analysed by flow cytometry using PI staining (FL3-A). (B) The quantitative percentage of cells in cell cycle distribution. (C) Immunoblots and band intensities of cyclin B1 and CDK1 following treatment with the indicated concentrations of nintedanib for 48 h. (D) Immunoblots and band intensities of cyclin B1 and CDK1 following treatment with 2 µM of nintedanib at different time courses. The data were presented as the mean ± SD of three experiments in all experiments. The result was normalised and statistically analysed in one-way ANOVA with Bonferroni post-test (*p < 0.05).

One of the main cellular consequences of p53 activation in proliferating cells is to induce cell cycle arrest. Consequently, the cell cycle distribution was assessed in HCT116 cells when treated with nintedanib (Figures 9A,B). MDM2 inhibitor nutlin-3 (3 µM) significantly induces G2 cell cycle arrest. Likewise, G2/M phase arrest is observed with increased HCT116 cells from 12.9% to 31.0% upon treatment with 0.2 µM of nintedanib. The percentage of G2 phase cells is further increased to 35.6% by 1 µM of nintedanib. These results demonstrate that nintedanib can induce significant cell cycle arrest in the G2/M phase.

**Figure 10.**
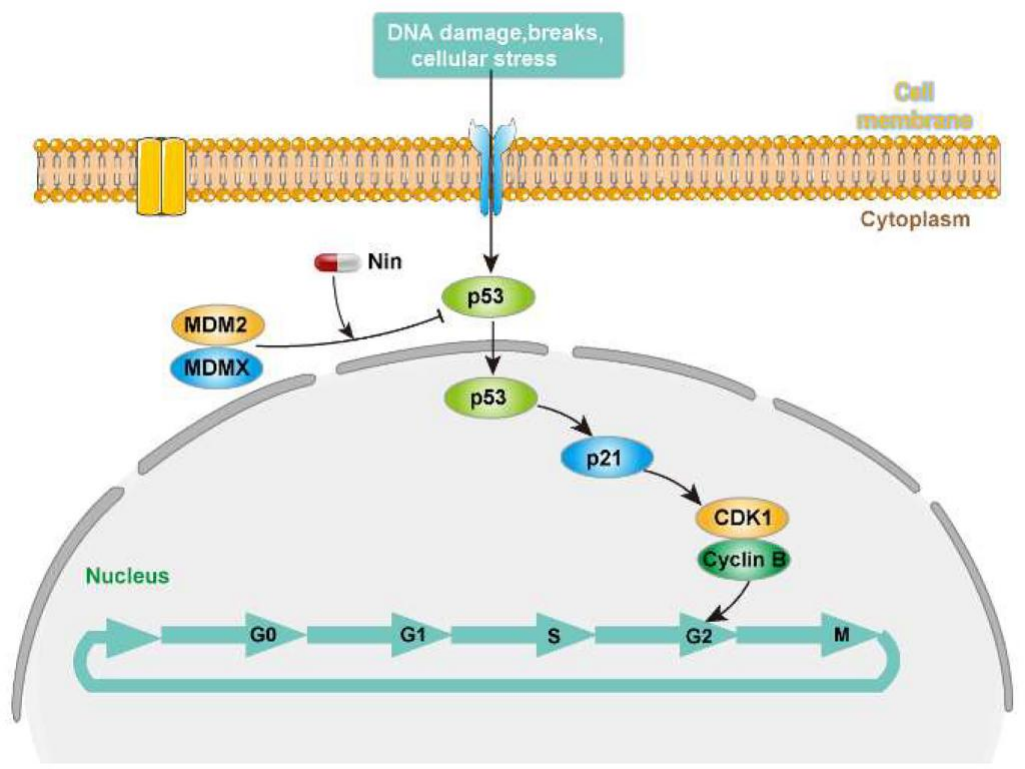
The proposed pathway for the antitumor effect of nintedanib.

The cell cycle is a process of sequential activation and inactivation of CDK proteins, a family of serine/threonine protein kinases. The maintenance of G2 to M blockade is p53-dependent and involves its transcriptional targets, such as p21, which mediates the cell cycle arrest by directly binding cyclin B1/CDK1 complex to ensure their inactivation^27^. As demonstrated above, nintedanib could upregulate p21 and induce the G2/M cell cycle arrest. Therefore, we profiled the cyclin B1 and CDK1 levels by western blot after treating cells with nintedanib. Nintedanib downregulates cyclin B1 and CDK1 dose-dependently (Figure 9C). The time-dependency of downregulation was also validated by treating cells with 2 µM of nintedanib at different time courses (Figure 9D). Overall, all these results suggest that nintedanib disrupts the p53-MDM2/MDMX interaction and reinitiates the transcriptional function of p53, which further induces G2/M phase arrest in HCT116 cells via the downregulation of cyclin B1 and CDK1 (Figure 10).

### Structural minimisation of nintedanib

**Figure 11.**
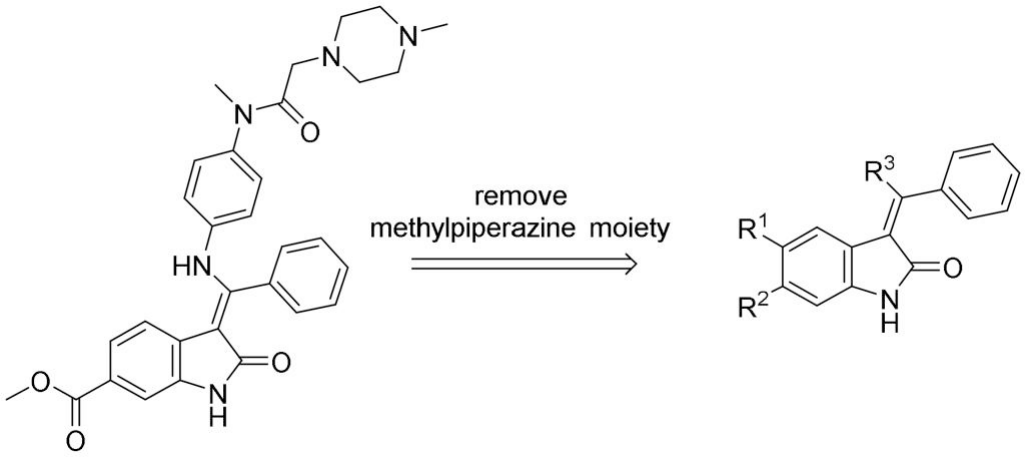
Minimisation of the chemical structure of nintedanib.

**Scheme 1.**
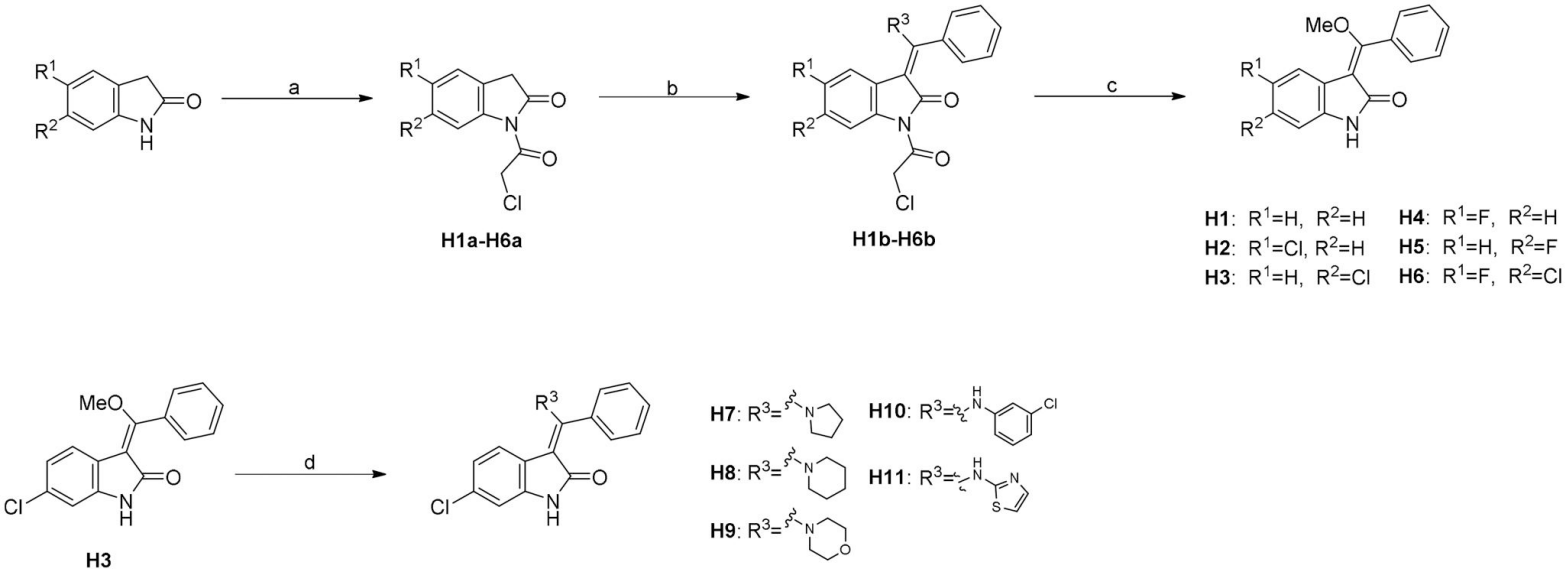
Synthesis of H1-H11. Reagents and conditions: (a) chloroacetic anhydride, toluene, reflux; (b) trimethyl orthobenzoate, acetic anhydride, toluene, 110 °C; (c) KOH, MeOH, 60 °C; (d) amine derivatives, MeOH, DMF, and reflux.

After an extensive mechanistic investigation, nintedanib was identified as a dual inhibitor of MDM2/MDMX. Molecular docking revealed that the methylpiperazine moiety stretched further into the Leu26 pocket, causing a steric clash against MDM2. Therefore, we envisaged that minimising nintedanib might increase the binding potency to MDM2 or MDMX. As shown in Figure 11, the benzylindolone core structure of nintedanib was maintained, and methylpiperazine moiety was removed. A series of derivatives (H1-H11) were designed and synthesised (Scheme 1).

The synthesis of H1-H11 was outlined in Scheme 1. Commercially available 2,3-dihydro-1*H*-indol-2-one reacted with chloroacetic anhydride in toluene to give **H1a-H6a**, which were then converted into **H1b-H6b** in the presence of trimethyl orthoformate and acetic anhydride in toluene. Under the basic condition, **H1b-H6b** were hydrolysed to afford **H1-H6** feasibly. Finally, compounds **H7-H11** were achieved by the nucleophilic attack of **H3** by a panel of amines.

### In vitro biological evaluation of nintedanib and its derivatives

For the rapid screening and comparison of nintedanib and its derivatives, the fluorescence polarisation (FP) method was conducted to measure the protein binding inhibition rate at 1 μM concentration of compounds for MDM2 and MDMX. The results were summarised in Table 3.

**Table 3.** Protein binding inhibition rate of each compound for MDM2 and MDMX.

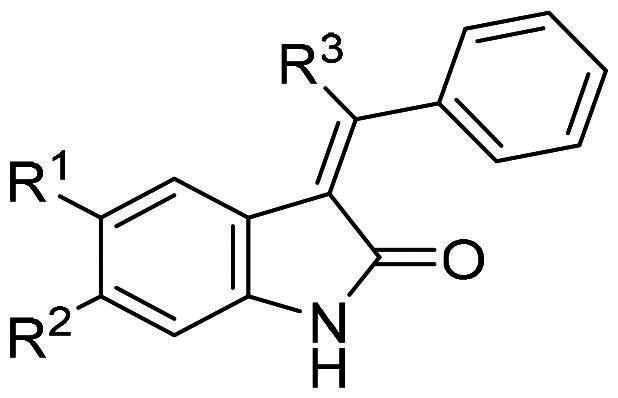					
Compound	*R^1^*	*R^2^*	*R^3^*	**Protein binding inhibition at 1 μM(%)** a	
				MDM2	MDMX
Nintedanib	—	—	—	60.6 ± 3.1	76.5 ± 5.5
H1	H	H	OMe	31.4 ± 0.6	18.2 ± 2.7
H2	Cl	H	OMe	38.7 ± 1.0	21.3 ± 2.1
H3	H	Cl	OMe	72.8 ± 5.6	57.9 ± 6.1
H4	F	H	OMe	42.1 ± 3.3	15.4 ± 2.3
H5	H	F	OMe	56.8 ± 1.9	16.8 ± 3.1
H6	F	Cl	OMe	50.3 ± 2.5	13.1 ± 2.7
H7	H	Cl	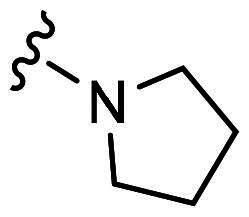	52.6 ± 2.1	34.1 ± 0.6
H8	H	Cl	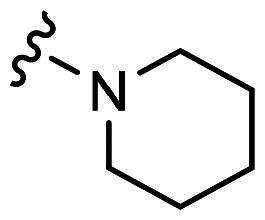	18.7 ± 0.8	25.6 ± 2.2
H9	H	Cl	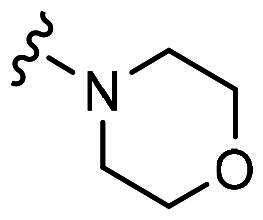	19.3 ± 1.5	21.2 ± 4.5
H10	H	Cl	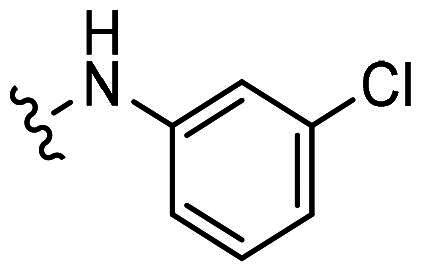	16.5 ± 2.8	23.8 ± 3.1
H11	H	Cl	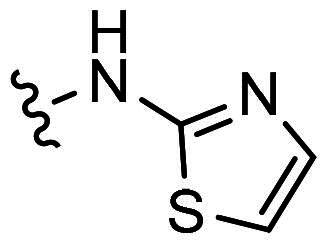	48.4 ± 4.6	15.4 ± 1.7

^a^ The protein binding inhibition at 1 μM of the compound was evaluated in a competitive fluorescence polarisation assay with the corresponding proteins in triplicate. Data are presented as mean ± SD.

At 1 μM concentration, nintedanib displaced the fluorescent p53 peptide with a 60% and 76% ratio for MDM2 and MDMX, respectively. When the benzamide methylpiperazine part was removed to give **H1**, the binding ability to both proteins was dramatically decreased. Interestingly, introducing a halogen group to indolone enhances the binding, especially for MDMX. **H2** and **H4** with chloro- and fluoro- at the 5-position of indolone behave with a higher inhibition ratio than parent **H1**. Furthermore, compared to 5-halo compounds (**H2** and **H4**), the 6-halogenated compounds **H3** and **H6** are more potent, with **H3** as the most potent among **H1-H6**.

With **H3** in hand, we also prepared a set of compounds with amine replacing the methoxy group, affording compounds **H7-H11**. **H7** with a 5-membered pyrrolidine ring displayed a slightly decreased binding ability to MDM2 and MDMX. The affinity was significantly lost when switched to a 6-membered piperidine ring (**H8**) or morpholine ring (**H9**). Moreover, the substituted aniline (**H10**) was also detrimental to the binding. However, the 2-amino thiazole substitution could somewhat rescue the binding to MDM2 but not MDMX.

To summarise, **H3** was screened out from this small set of compounds. The structural relationship analysis (SAR) shows that electro-withdrawing halogen on the 6-position of indolone favours the binding, particularly for MDM2. For the *R*^3^ group, the smaller substituents, such as methoxy and pyrrolidine, demonstrate less damage to the protein binding, which might be attributed to steric decompression when binding the targets.

To evaluate whether the nintedanib derivatives have the potential antiproliferative activity, CCK-8 assay was performed in SJSA-1, SHSY-5Y, and HCT116, with p53-deleted HCT116 cell as a negative control. The results were summarised in Table 4.

**Table 4.** Antiproliferative activity of nintedanib derivatives in different cells.

	**IC_50_ (μM)** a			
Compounds	SJSA-1	SHSY-5Y	HCT116	HCT116 p53-/-
Nutlin-3	2.56 ± 2.04	19.44 ± 1.17	0.99 ± 2.08	10.43 ± 15.32
Nintedanib	1.11 ± 1.69	0.08 ± 0.22	0.31 ± 1.89	8.35 ± 2.04
H2	9.56 ± 1.30	5.37 ± 1.01	9.24 ± 2.25	11.24 ± 9.25
H3	1.66 ± 1.83	4.24 ± 0.31	3.40 ± 4.28	28.34 ± 3.03
H4	8.59 ± 0.94	>10	>10	—
H5	7.16 ± 1.05	4.69 ± 0.58	8.51 ± 3.90	14.23 ± 1.41
H6	8.78 ± 1.65	6.11 ± 1.25	7.10 ± 4.38	10.16 ± 20.21
H7	6.66 ± 0.16	9.23 ± 3.09	6.18 ± 1.02	13.89 ± 2.94
H8	7.59 ± 0.82	9.14 ± 2.12	6.24 ± 2.77	29.45 ± 1.04
H9	7.45 ± 1.69	>10	7.24 ± 8.02	—

^a^ The compounds’ cell growth inhibition (IC_50_) was measured in the corresponding cells by CCK-8 assay in triplicate. Data are presented as mean ± SD.

As expected, none of the compounds show evident cell growth inhibition in p53-deleted HCT116 cells (IC_50_ >10 μM). In p53 wild-type cells, most compounds exhibit moderated antiproliferative activity, although it is inferior to nintedanib. Among them, compound **H3**, with the highest binding ability to MDM2/MDMX, displays the most potent cell growth inhibition with IC_50_ values of 1.1, 4.2, and 3.4 μM in SJSA-1, SHSY-5Y, and HCT116 cell, respectively. Of note, **H3** is more potent than nutlin-3 in SJSA-1 and SHSY-5Y cells.

**Figure 12.**
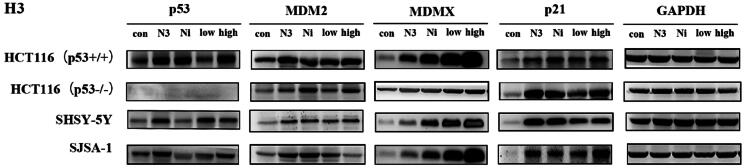
Effect of compound H3 on the level of related proteins. N3: nutlin-3 (5 μM); Ni: nintedanib (5 μM); low: H3 (5 μM); high: H3 (10 μM).

Since **H3** has shown potential antiproliferation in p53 wild-type cells, an immunoblot experiment was employed to examine whether **H3** worked via the disruption of MDM2/MDMX-p53 interaction (Figure 12). When cells were incubated with nutlin-3 (5 μM), nintedanib (5 μM), and H3 (5 μM or 10 μM) for 48 h, respectively, the levels of both p53 and MDM2 were significantly upregulated, as well as the level of p21. In contrast, in p53-deleted cells, the activation was less pronounced. Collectively, by structural minimisation, **H3** was validated as a dual inhibitor of MDM2/MDMX, which can disrupt MDM2/MDMX-p53 and show antiproliferative activity in p53 wild-type cells. Nonetheless, the potency needs to be further increased, and more comprehensive structural optimisation is still ongoing in our lab.

## Conclusion

To our knowledge, our studies are the first to unveil that nintedanib activates the function of p53 by disrupting the p53-MDM2/MDMX interactions. Compared with MDM2 inhibitors, dual blockage of MDM2/MDMX is more promising, although with no marketed drugs so far. In this work, we demonstrate that nintedanib induces the upregulation of p53 and its downstream transcriptional targets, further inhibiting cell growth and initiating the G2 cell cycle arrest. Moreover, our findings state that nintedanib can be repurposed as an MDM2/MDMX dual inhibitor. In addition, 11 compounds were designed and synthesised to gain insights into SAR, of which H3 exhibits moderate potency in p53 wild-type cells and can be the lead compound for further development.

## Experimental section

### Reagents and materials

Nintedanib (>98%) was purchased from KAIWEI CHEMICAL Co., Ltd. (Shanghai, China). The nintedanib solution was prepared for each experiment in DMSO (Beijing Solarbio Science & Technology Co., Ltd.). TRIzol reagent, DEPC-treated water, RNase, DNase, and DNA Away H_2_O were obtained from Vazyme Biotech Co., Ltd. Nutlin-3 was purchased from Absin. RIPA lysis buffer and BCA Protein kit were purchased from YIFEIXUE BIOTECH (Nanjing, China). The primary antibodies were used in the study, including anti-MDM2, anti-CDK1, anti-cyclinB, anti-BAX, anti-p21, anti-β-actin, and anti-GAPDH antibodies (ABclonal Technology Co., Ltd. Wuhan, China); anti-p53 antibody (Cohesion Biosciences Co., Ltd.); anti-MDMX antibody (Proteintech Group, Wuhan, China). The secondary antibodies HRP-labelled goat anti-rabbit IgG (H + L) and HRP-labelled goat anti-mouse IgG were from ABclonal Technology Co., Ltd. (Wuhan, China). DMEM-H cell culture medium, foetal bovine serum (FBS), 1% penicillin/streptomycin, and 0.25% trypsin were purchased from Procell Life Science & Technology Co., Ltd. (Wuhan, China). MDM2 (residues 1–110) and MDMX (residues 1–110) were expressed and purified by GenScript (Nanjing, China). 5-FAM labelled PMDM6-F (abs45157068) was purchased from Absin.

### Structure-based virtual screening of potential dual inhibitors

The FDA-approved drugs database (1355 purchasable compounds) was selected for screening. Virtual screening of the ZINC database was performed using the molecular docking software Schrödinger 2019. Compounds were minimised energetically by using MMFF94 with 5000 iterations and a minimum RMS gradient of 0.05. The protein structures MDM2 (PDB ID: 3VBG) and MDMX (PDB ID: 3U15) were obtained from the Protein Data Bank. Proteins were prepared by using the Protein Preparation Wizard module in Schrödinger 2019. Water was removed and polar hydrogens were added. In addition, the protonated state of all residues was assumed to be involved, such as His being protonated to HID, and Asp, Arg, Glu, and Lys being pre-treated as the deprotonated state. Receptor grids were generated using Receptor Grid Generation, and binding sites were generated within a 10 Å radius of the ligand centre, including several key amino acid residues. Finally, using the Glide module, molecular docking was performed in Standard Precision (SP) and Extra Precision (XP) modes. The other parameters are default values. A consensus score (CS) for each compound was generated. The CS score represents compound potential as a dual MDM2 and MDMX inhibitor. Here, the CS of compound c is defined as

$$CS(c)=R_{\mathrm{MDM}2}(c)+R_{\mathrm{MDMX}}(c)$$

where *R_MDM2_(c)* and *R_MDMX_(c)* are the ranking score of compound c in MDM2 and MDMX, respectively. The compounds were then manually filtered to remove the compounds that the structural centre of the complex did not fit well with the active centre. Schrodinger 2019 was used to dock nintedanib to the pocket site of potential targets to detect the possible binding modes. The top-scoring pose was used for the following MD simulation. The binding patterns of the docked pose were visualised by PyMol 1.1 (Schrodinger, New York).

### Molecular dynamic simulation

The proteins and ligands from the molecular docking results were separated, and the ligand force field files were generated using the antechamber tool in the Ambertools software, and the ligand force field files were converted to gromacs force field files by the acpype software tool. GAFF force field was used for ligand, AMBER14SB force field and TIP3P water model were used for protein, protein and ligand files were merged to construct the simulation system of the complex.

The molecular dynamics simulations were carried out using the Gromacs2022 program under constant temperature and pressure and periodic boundary conditions. In the MD simulations, all hydrogen bonds were constrained using the LINCS algorithm with an integration step of 2 fs. Electrostatic interactions were calculated using the (Particle-mesh Ewald) PME method with a cut-off value of 1.2 nm. The electrostatic interactions are calculated by the (Particle-mesh Ewald) PME method with a cut-off value of 1.2 nm, and the non-bonding interactions have a cut-off value of 10 Å, which is updated every 10 steps. The simulation temperature was controlled by the V-rescale temperature coupling method at 298 K, and the pressure was controlled by the Berendsen method at 1 bar. 100 ps NVT and NPT equilibrium simulations were carried out at 298 K. 100 ns MD simulations were carried out for the complex system, and the conformation was saved every 10 ps. After the simulations were completed, the trajectories were analysed and the complexes were subjected to MM/GBSA binding free energy analysis using the g_mmpbsa program.

### Binding-free energy calculation

The binding free energy based on the protein-ligand complex generated by the MD procedure was calculated using a molecular mechanics-generalised Born surface area (MM/GBSA) module. The binding-free energy was calculated as follows:

$$\begin{array}{c} {\Delta G}_{\mathrm{bind}}={\text{ΔG}}_{\mathrm{complex}}\;–\;({\text{ΔG}}_{\mathrm{receptor}}+\;{\text{ΔG}}_{\mathrm{ligand}})\\ ={\text{ΔE}}_{\mathrm{vdw}}+{\text{ΔE}}_{\mathrm{elec}}{+\Delta G}_{\mathrm{pol}}+{\text{ΔG}}_{\mathrm{nonpol}} \end{array}$$

In the formula, ΔE_vdw_ means van der Waals effect, ΔE_elec_ means electrostatic interaction. ΔE_pol_ means polar solvation energy, ΔE_nonpol_ means non-polar solvation. The Generalised Born (GB) continuum model calculated the former, and the latter was estimated by the solvent-accessible surface area (SASA) approach, GSA = 0.0072 × ΔSASA. This study ignores the entropy change due to the high consumption of calculation resources and low precision^28^^,^^29^.

### Microscale thermophoresis (MST) binding assay

The binding affinities of nintedanib to MDM2 and MDMX were determined using an optimised microscale thermophoresis (MST) binding assay. The diluted his-tagged MDM2 (1–110) and MDMX (1–110) were labelled using a labelling kit (RED-tris-NTA 2nd Generation, NanoTemper). The labelled proteins were then mixed with a serial dilution of nintedanib (ranging from 0.0122 to 200 μM) in assay buffer (50 nM NaH_2_PO_4_/Na_2_HPO_4_ pH 7.5, 200 mM NaCl, 5% glycerol, and 0.001% Tween). Approximately 4–6 μL of each sample was loaded in a fused silica capillary (NanoTemper Technologies). Measurements were performed at room temperature in a Monolith NT.115 Pico instrument at a constant LED power of 60–90% and MST power of 20% and 40%. The laser-on time was 20 s for each measurement. Measurements were performed repeatedly on independent protein preparations to ensure reproducibility. The data were analysed by plotting nintedanib concentrations against ligand-induced fluorescence changes (change in raw fluorescence on the y-axis). Curve fitting was performed using Prism 8 (GraphPad Software), giving the *K*_d_ values with a 95% confidence level.

### Cell culture and treatment

Human cells HCT116 (RIDD: CVCL_0291; catalogue number: CCL-247), MCF-7 (RIDD: CVCL_0031; catalogue number: SCSP-531), MD-AMB-453 (RIDD: CVCL_0418; catalogue number: TCHu233), and SH-SY5Y (RIDD: CVCL_0019; catalogue number: SCSP-5014) were obtained from the cell bank of the Chinese Academy of Sciences (Shanghai, China). Human cells HCT116 p53^-/-^ (CVCL_HD97; catalogue number: YS4059C) and SJSA-1 (CVCL_1697; catalogue number: CBP60236) were purchased from Bairui Technology Co., Ltd. (Nanjing, China). All cells were cultured in DMEM-H medium, while SJSA-1 cells were cultured in RPMI-1640 medium supplemented with 10% FBS and 1% penicillin/streptomycin at 37 °C under a humidified atmosphere of 5% CO_2_ condition.

### Cell viability assay

Cell viability was evaluated by Cell Counting Kit-8 (GlpBio Technology, USA). Cells were seeded in a 96-well plate at a density of 5 × 10^3^ cells. After being incubated for 24 h, cells were treated with different concentrations of nintedanib and incubated at 37 °C under a humidified 5% CO_2_ atmosphere for 48 h. Cell Counting Kit-8 (1/10 volume) was added directly to the cells in the culture medium. The plate was mixed thoroughly and incubated for 1 to 4 h at 37 °C until the colour turned orange. The absorbance was measured at 450 nm with a SpectraMax i3x microplate reader (Molecular Devices). The absorbance was normalised, and the IC_50_ was calculated by non-linear regression analysis using GraphPad Prism 8 software.

### Cell migration assay

Cell migration was examined using a wound healing assay in a six-well plate. SH-SY5Y cells were cultured to create a confluent monolayer; then the cell monolayer was scraped in a straight line with a 200 μL pipet tip to create a “scratch.” Cells were washed with PBS three times and treated with the indicated concentrations of the drugs for 24 and 48 h, respectively. The scratch closure was monitored and imaged in 24 intervals by a microscope. Image J software was used to analyse the repair area. The repair rate was calculated using the formula: repair rate = (the empty area at 0 h − the empty area at 24 or 48 h)/the empty area at 0 h × 100%.

### Cell cycle analysis by flow cytometry

The cell cycle was evaluated by flow cytometry using PI staining (Beyotime Biotechnology Co Ltd.). The cells were plated at a density of 5 × 10^5^ in a six-well plate. The cells were collected post-treatment and fixed in ice-cold 70% ethanol overnight at −20 °C. The cells were centrifuged and washed with PBS; then cells were stained with PI solution containing RNase at room temperature for 30 min. Subsequently, the data were acquired using a Gallios flow cytometer (Beckman Coulter Life Sciences Co., Ltd.). A minimum of 1 × 10^5^ cells from each sample were analysed regarding DNA content, and the percentage of cells in each cell cycle phase was quantified. Analysis of the data was done by Modfit software (Verity Software House Inc., Topsham, ME) for the proportions of different cell cycle phases.

### RT-qPCR analysis

Cells were treated with the indicated concentration of nintedanib in different time courses. The mRNA levels of the related genes were determined by quantitative RT-PCR and expressed as a fold change compared with the untreated control. Total RNA was extracted using TRIzol reagent, and the reverse transcription reaction was performed using HiScriptIIQ RT Supermix (Vazyme Biotech Co., Ltd.). qPCR was conducted on a Quantstudio 6 Flex Applied Biosystems (Thermo Fisher Scientific, USA) using ChamQ Universal SYBER qPCR Master Mix (Vazyme Biotech Co., Ltd.). The expression of the target gene was analysed by the comparative CT (ΔΔCT) method. Primers were from Origene, and the sequences are shown below.

**Table:** 

Gene	Forward sequence	Reverse sequence
TP53	5′-CCTCAGCATCTTATCCGAGTGG-3′	5′-TGGATGGTGGTACAGTCAGAGC-3′
MDM2	5′-TGTTTGGCGTGCCAAGCTTCTC-3′	5′-CACAGATGTACCTGAGTCCGATG-3′
MDMX	5′-CAGCAGGTGCGCAAGGTGAA-3′	5′-CTGTGCGAGAGCGAGAGTCTG-3′
BAX	5′-TCAGGATGCGTCCACCAAGAAG-3′	5′-TGTGTCCACGGCGGCAATCATC-3′
CDKN1A	5′-AGGTGGACCTGGAGACTCTCAG-3′	5′-TCCTCTTGGAGAAGATCAGCCG-3′
GAPDH	5′-GTCTCCTCTGACTTCAACAGCG-3′	5′-ACCACCCTGTTGCTGTAGCCAA-3′

### Immunofluorescence assay

Cells were seeded on coverslips, washed twice with PBS (phosphate-buffered saline), and fixed with 4% paraformaldehyde (PFA) solution at room temperature for 20 min. The coverslips were blocked using 3% BSA (PBS buffer) for 30 min, then incubated with p53 primary antibody overnight (1: 200, Wuhan Servicebio Technology CO., LTD). After washing with PBS three times, the coverslips were incubated with Sulfo-Cyanine3 (CY3) labelled Goat Anti-Rabbit lgG (H + L) (1:300, Wuhan Servicebio Technology CO., LTD) for 1 h at room temperature in the dark. Then cell nuclei were counterstained with DAPI (Solarbio, China), and cells were visualised under NIKON ECLIPSE C1 (NIKON, Japan) fluorescence microscope.

### Immunoblotting assay

After being treated with the indicated drugs, cells were collected and lysed with RIPA buffer supplemented with protease inhibitors. The supernatant was collected after centrifugation at 12,000 rpm at 4 °C for 20 min. Protein concentrations were determined using the BCA Protein Assay Kit. A total of 20 μg proteins were resolved by 10% SDS-PAGE under reducing conditions and transferred to polyvinylidene difluoride (PVDF) membranes (Merck Millipore Ltd. Tullagreen, Carrigtwohill, Co. Cork IRL Rev.02/18). Membranes were blocked in a 5% BSA TBS-T buffer. Proteins were probed with the indicated primary antibodies (1:1000) at 4 °C overnight (the detailed antibody information seen below). The membrane was then washed and incubated with HRP-linked secondary antibodies (1:10000) for 1 h at room temperature. After washing, the membrane was incubated with ECL reagent and visualised by Tanon-4200 (Tanon. Co., Ltd. Shanghai, China). The band intensity was quantified by Image J software (National Institutes of Health, Bethesda, MD, USA).

Primary antibodies: p53 (rabbit, Cat No. A0263, ABclonal); MDM2 (mouse, Cat No. 66511–1-lg, Proteintech); MDMX (mouse, Cat No. 17914–1-AP, Proteintech); p21 (rabbit, Cat No. A1483, ABclonal); Bax (rabbit, Cat No. A19684, ABclonal); Cyclin B1 (rabbit, Cat No. A2056, ABclonal); CDK1 (rabbit, Cat No. A14715, ABclonal); GAPDH (rabbit, Cat No. AC001, ABclonal); and β-actin (rabbit, Cat No. AC038, ABclonal). Secondary antibodies: HRP Goat Anti-Rabbit IgG (H + L) (rabbit, Cat No. AS014, ABclonal); HRP Goat Anti-Mouse IgG (H + L) (mouse, Cat No. AS003, ABclonal).

### Fluorescent polarisation binding assay

The FP assay was performed in black 384-well plates at a final volume of 100 μL per well. Assay plates were prepared by addition of 35 μL of assay buffer (100 mM PBS pH 7.5, 100 μg/mL bovine-γ-globulin, 0.01% TX-100) containing 40 nM MDM2 (or 1280 nM MDMX) and 35 μL of 5-FAM labelled PMDM6-F (15 nM in assay buffer) (AnaSpec) to each well. For testing, a solution of small molecules was added (25 μL) by direct addition to the assay plate, giving a final concentration of 1 μM. The assay mixture was incubated for 0.5 h at room temperature. The FP values in millipolarization units (mP) were read on Molecular Devices (Thermo) with 485 nm excitation and 535 nm emission filters at room temperature. Technical triplicate data was normalised to the positive (100% inhibition) and negative (0% inhibition) controls on the corresponding row of the 384-well plate. The inhibition rate was calculated using the equation: the percentage inhibition = 100 × (sample result – negative control)/(positive control mean – negative control).

### Compound synthesis

Unless otherwise noted, all reagents and dry solvents were purchased from commercial sources and used without further purification. NMR spectra were recorded on a Brüker Avance III, 500 MHz instrument in ppm relative to the residue solvent signals of DMSO (δ = 2.51 ppm for ^1^H NMR and 40.1 ppm for ^13^C NMR). Signal splitting patterns were described as singlet (s), doublet (d), triplet (t), or multiplet (m), with coupling constants (J) in hertz. High-resolution mass spectra (HRMS) were performed on the AB Sciex QTOF 5600 mass spectrometer with an electrospray ionisation source. The purity of the compounds for the biological test was confirmed above 95% using a Waters e2695 (Separations Module) liquid chromatograph with a Hedera ODS-2 C18 column (5 μm 4.6 × 250 mm).

#### The general method for the synthesis of H1b-H6b

A solution of 2,3-dihydro-1H-indol-2-one (0.665 g, 5 mmol) in toluene (5 ml) was placed in a round-bottom flask, followed by the addition of chloroacetic anhydride (1.275 g, 7.5 mmol). The reaction mixture was then refluxed for 3 h. The reaction was cooled down to 80°Cand methylcyclohexane (2.5 ml) was added to form the precipitate. The precipitate was filtered and washed with cold methanol, yielding compounds H1a-H6a. To a solution of H1a-H6a (5 g, 23.9 mmol) in toluene (30 ml), acetic anhydride (7.9 ml, 83.65 mmol) was added at r.t. The reaction mixture was heated to 110 °C, and trimethyl orthobenzoate (10.4 g, 57.4 mmol) was added. The mixture was stirred for 3 h before the removal of the solvent. Another 4 ml of toluene was added, and the mixture was cooled down to 5 °C. The formed precipitate was filtered and washed with toluene and ethyl acetate to give H1b-H6b as yellow solids.

*1–(2-chloroacetyl)-3-(methoxy(phenyl)methylene)indolin-2-one (H1b):* Yellow solid; yield: 17.2%; HPLC purity: 99.84%.^1^H NMR (500 MHz, DMSO-*d*6): δ 8.18 − 8.15 (m, 1H), 8.00 (dd, *J* = 7.5, 1.2 Hz, 1H), 7.59 − 7.55 (m, 3H), 7.50 − 7.46 (m, 2H), 7.31 (dtd, *J* = 13.8, 7.5, 6.3 Hz, 2H), 4.85 (s, 2H), 3.72 (s, 3H).^13^C NMR (126 MHz, DMSO-*d6*): δ 171.28, 167.03, 166.78, 136.16, 130.95, 130.80, 129.33, 129.06, 127.59, 125.34, 124.51, 123.39, 115.37, 105.06, 58.45, and 47.02. ESI-HRMS(*m/z*) calculate for C_18_H_15_ClNO_3_^+^ [M + H]^+^ 328.0735 found 328.0725.

*5-chloro-1–(2-chloroacetyl)-3-(methoxy(phenyl)methylene)indolin-2-one (H2b):* Yellow solid; yield: 28.2%; HPLC purity: 99.80%.^1^H NMR (500 MHz, DMSO-*d6*): δ 8.17 (d, *J* = 8.7 Hz, 1H), 7.97 (d, *J* = 2.3 Hz, 1H), 7.64 − 7.59 (m, 3H), 7.51 (dd, *J* = 7.6, 1.6 Hz, 2H), 7.41 (dd, *J* = 8.7, 2.3 Hz, 1H), 4.85 (s, 2H), and 3.79 (s, 3H).^13^C NMR (126 MHz, DMSO-*d6*): δ 172.79, 167.00, 166.21, 134.73, 131.01, 130.57, 129.43, 129.19, 129.11, 127.05, 126.33, 122.62, 116.78, 104.10, 58.86, and 46.94. ESI-HRMS(*m/z*) calculate for C_18_H_14_Cl_2_NO_3_^+^ [M + H]^+^ 362.0345 found 362.0337.

6*-chloro-1–(2-chloroacetyl)-3-(methoxy(phenyl)methylene)indolin-2-one (H3b):* Yellow solid; yield: 33.5%; HPLC purity: 99.90%.^1^H NMR (500 MHz, DMSO-*d6*): δ 8.20 (d, *J* = 2.1 Hz, 1H), 8.01 (d, *J* = 8.3 Hz, 1H), 7.64 − 7.59 (m, 3H), 7.52 (dd, *J* = 7.7, 1.8 Hz, 2H), 7.39 (dd, *J* = 8.3, 2.1 Hz, 1H), 4.88 (s, 2H), and 3.78 (s, 3H).^13^C NMR (126 MHz, DMSO-*d6*): δ 172.08, 167.23, 166.35, 136.88, 131.39, 130.97, 130.64, 129.25, 129.10, 125.09, 124.44, 123.46, 115.35, 104.27, 58.70, and 46.93. ESI-HRMS(*m/z*) calculate for C_18_H_14_Cl_2_NO_3_^+^ [M + H]^+^ 362.0345 found 362.0338.

*1–(2-chloroacetyl)-5-fluoro-3-(methoxy(phenyl)methylene)indolin-2-one (H4b):* Brown solid; yield: 18.9%; HPLC purity: 99.39%.^1^H NMR (500 MHz, DMSO-*d6*): δ 8.19 (dd, *J* = 8.9, 5.0 Hz, 1H), 7.79 (dd, *J* = 8.9, 2.8 Hz, 1H), 7.64 − 7.59 (m, 3H), 7.52 (dd, *J* = 7.7, 1.7 Hz, 2H), 7.20 (td, *J* = 9.2, 2.8 Hz, 1H), 4.87 (s, 2H), and 3.80 (s, 3H).^13^C NMR (126 MHz, DMSO-*d6*): δ 172.61, 166.87, 166.47, 160.75, 158.85, 132.38, 131.00, 130.58, 129.28, 129.23, 126.27, 116.70, 113.73, 110.27, 104.60, 58.79, and 46.89. ESI-HRMS(*m/z*) calculate for C_18_H_14_ClFNO_3_^+^ [M + H]^+^ 346.0641 found 346.0632.

*1–(2-chloroacetyl)-6-fluoro-3-(methoxy(phenyl)methylene)indolin-2-one (H5b):* Red solid; yield: 33.7%; HPLC purity: 99.68%.^1^H NMR (500 MHz, DMSO-*d6*): δ 8.00 (dd, *J* = 8.6, 6.0 Hz, 1H), 7.94 (dd, *J* = 10.5, 2.6 Hz, 1H), 7.60 − 7.55 (m, 3H), 7.48 (dd, *J* = 8.6, 2.6 Hz, 2H), 7.17 − 7.12 (m, 1H), 4.85 (s, 2H), 3.72 (s, 3H).^13^C NMR (126 MHz, DMSO-*d6*): δ 172.79, 167.00, 166.21, 134.73, 131.01, 130.57, 129.43, 129.20, 129.11, 127.05, 126.33, 122.63, 116.78, 104.11, 58.86, 46.95. ESI-HRMS(*m/z*) calculate for C_18_H_14_ClFNO_3_^+^ [M + H]^+^ 346.0641 found 346.0632.

*6-chloro-1–(2-chloroacetyl)-5-fluoro-3-(methoxy(phenyl)methylene) indolin-2-one (H6b):* White solid; yield: 11.4%; HPLC purity: 99.80%.^1^H NMR (500 MHz, DMSO-*d6*): δ 8.25 (d, *J* = 6.8 Hz, 1H), 7.93 (d, *J* = 9.6 Hz, 1H), 7.59 (d, *J* = 6.8 Hz, 3H), 7.51 − 7.48 (m, 2H), 4.83 (s, 2H), and 3.78 (s, 3H).^13^C NMR (126 MHz, DMSO-*d6*): δ 173.33, 167.07, 165.95, 131.16, 130.32, 129.23, 129.18, 129.13, 129.09, 128.77, 128.61, 128.33, 116.77, 59.03, 49.06, and 46.80. ESI-HRMS(*m/z*) calculate for C_18_H_13_Cl_2_FNO_3_^+^ [M + H]^+^ 380.0251 found 380.0240.

#### The general method for the synthesis of H1-H6

To a solution of H1b-H6b (0.8 g, 2.45 mmol) in methanol (4 ml) was added a solution of KOH (0.041 g, 0.735 mmol) in methanol (1.5 ml). The reaction was stirred at 60 °C for 30 min and then cooled to 0 °C. The mixture was stirred for another 2 h. The precipitate was filtered and washed with methanol to give H1-H6 as yellow solids.

*3-(Methoxy(phenyl)methylene) indolin-2-one (H1):* Yellow solid; yield: 32.7%; HPLC purity: 99.38%.^1^H NMR (500 MHz, DMSO-*d6*): δ 10.16 (s, 1H), 7.76 (d, *J* = 7.5 Hz, 1H), 7.52 − 7.48 (m, 3H), 7.45 − 7.41 (m, 2H), 7.12 (td, *J* = 7.5, 1.2 Hz, 1H), 6.95 (td, *J* = 7.6, 1.2 Hz, 1H), 6.78 (d, *J* = 7.6 Hz, 1H), and 3.61 (s, 3H).^13^C NMR (126 MHz, DMSO-*d6*): δ 168.26, 167.24, 139.84, 131.39, 130.26, 129.78, 128.67, 127.36, 123.77, 123.66, 121.13, 108.97, 108.09, and 57.61. ESI-HRMS(*m/z*) calculate for C_16_H_14_NO_2_^+^ [M + H]^+^ 252.1019 found 252.1012.

*5-chloro-3-(methoxy(phenyl)methylene) indolin-2-one (H2):* Yellow solid; yield: 40.2%; HPLC purity: 99.15%.^1^H NMR (500 MHz, DMSO-*d6*): δ 10.29 (s, 1H), 7.72 (d, *J* = 2.0 Hz, 1H), 7.55 − 7.49 (m, 3H), 7.43 (dd, *J* = 7.3, 2.0 Hz, 2H), 7.17 (dd, *J* = 8.2, 2.2 Hz, 1H), 6.78 (d, *J* = 8.2 Hz, 1H), and 3.65 (s, 3H).^13^C NMR (126 MHz, DMSO-*d6*): δ 168.50, 167.06, 162.87, 160.95, 141.34, 131.20, 130.33, 129.77, 128.70, 120.01, 107.28, 97.13, and 57.66. ESI-HRMS(*m/z*) calculate for C_16_H_13_ClNO_2_^+^ [M + H]^+^ 286.0629 found 286.0621.

*6-chloro-3-(methoxy(phenyl)methylene) indolin-2-one (H3):* Yellow solid; yield: 45.8%; HPLC purity: 99.47%.^1^H NMR (500 MHz, DMSO-*d6*): δ 10.34 (s, 1H), 7.78 (d, *J* = 8.1 Hz, 1H), 7.57 − 7.52 (m, 3H), 7.49 − 7.46 (m, 2H), 7.03 (dd, *J* = 8.1, 2.0 Hz, 1H), 6.83 (d, *J* = 2.0 Hz, 1H), and 3.67 (s, 3H).^13^C NMR (126 MHz, DMSO-*d6*): δ 168.19, 168.12, 141.04, 131.31, 131.07, 130.43, 129.69, 128.73, 124.77, 122.60, 120.79, 108.91, 107.04, and 57.84. ESI-HRMS(*m/z*) calculate for C_16_H_13_ClNO_2_^+^ [M + H]^+^ 286.0629 found 286.0619.

*5-fluoro-3-(methoxy(phenyl)methylene) indolin-2-one (H4):* Yellow solid; yield: 25.6%; HPLC purity: 99.68%.^1^H NMR (500 MHz, DMSO-*d6*): δ 10.16 (s, 1H), 7.69 (p, *J* = 3.3 Hz, 1H), 7.55 − 7.48 (m, 4H), 7.44 (dd, *J* = 7.6, 1.9 Hz, 2H), 6.95 (ddd, *J* = 9.8, 8.7, 3.6 Hz, 1H), 6.76 (ddd, *J* = 13.0, 8.7, 3.6 Hz, 1H), 3.65 (d, *J* = 3.3 Hz, 3H).^13^C NMR (126 MHz, DMSO-*d6*): δ 168.60, 168.22, 136.03, 131.01, 130.46, 129.70, 128.72, 124.82, 113.40, 110.79, 109.34, 58.52, 57.90. ESI-HRMS(*m/z*) calculate for C_16_H_13_FNO_2_^+^ [M + H]^+^ 270.0925 found 270.0916.

*6-fluoro-3-(methoxy(phenyl)methylene) indolin-2-one (H5)*: Yellow solid; yield: 22.6%; HPLC purity: 99.32%.^1^H NMR (500 MHz, DMSO-*d6*): δ 10.30 (s, 1H), 7.75 (dd, *J* = 8.4, 5.8 Hz, 1H), 7.56 − 7.40 (m, 5H), 6.78 − 6.69 (m, 1H), 6.59 (dd, *J* = 9.3, 2.4 Hz, 1H), and 3.61 (s, 3H).^13^C NMR (126 MHz, DMSO-*d6*): δ 168.50, 167.06, 162.87, 160.95, 141.34, 131.20, 130.33, 129.77, 128.70, 124.88, 120.03, 120.01, 107.28, 97.13, and 57.66. ESI-HRMS(*m/z*) calculate for C_16_H_13_FNO_2_^+^ [M + H]^+^ 270.0925 found 270.0916.

*6-chloro-5-fluoro-3-(methoxy(phenyl)methylene) indolin-2-one (H6):* Yellow solid; yield: 22.1%; HPLC purity: 99.17%.^1^H NMR (500 MHz, DMSO-*d6*): δ 10.29 (s, 1H), 7.69 (d, *J* = 9.8 Hz, 1H), 7.55 − 7.49 (m, 3H), 7.46 − 7.42 (m, 2H), 6.87 (d, *J* = 6.3 Hz, 1H), and 3.67 (s, 3H).^13^C NMR (126 MHz, DMSO-*d6*): δ 169.38, 167.96, 153.92, 152.04, 136.66, 130.74, 129.62, 128.75, 123.78, 117.18, 111.82, 109.61, 106.91, and 58.10. ESI-HRMS(*m/z*) calculate for C_16_H_12_ClFNO_2_^+^ [M + H]^+^ 304.0535 found 304.0521.

#### The general method for the synthesis of H7-H11

To a solution of H3 (0.285 g, 1 mmol) in a mixture solvent of DMF and methanol (v/v: 1/3, 4 ml), amine (1 mmol) was added at r.t. The reaction mixture was then refluxed for 2 h, followed by a stirring at 0 °C for another 2 h. The formed precipitate was filtered and recrystallized from methanol, giving compounds H7-H11 as yellow solids.

*6-chloro-3-(phenyl(pyrrolidin-1-yl)methylene)indolin-2-one (H7)*: Yellow solid; yield: 27.5%; HPLC purity: 99.44%.^1^H NMR (500 MHz, CDCl_3_): δ 8.70 (s, 1H), 7.58 − 7.48 (m, 3H), 7.43 − 7.38 (m, 2H), 6.85 (d, *J* = 2.0 Hz, 1H), 6.46 (dd, *J* = 8.4, 2.0 Hz, 1H), 5.36 (d, *J* = 8.4 Hz, 1H), 3.75 (d, *J* = 6.7 Hz, 4H), and 2.03 (s, 4H).^13^C NMR (126 MHz, DMSO-*d6*): δ 172.63, 166.89, 166.49, 160.77, 158.87, 132.40, 131.02, 130.60, 129.25, 129.11, 126.29, 116.72, 113.75, 110.29, 104.62, 58.81, and 46.91. ESI-HRMS(*m/z*) calculate for C_19_H_18_ClN_2_O^+^ [M + H]^+^ 325.1097 found 325.1086.

6*-chloro-3-(phenyl(piperidin-1-yl)methylene)indolin-2-one (H8):* Yellow solid; yield: 44.8%; HPLC purity: 99.09%.^1^H NMR (500 MHz, DMSO-*d6*): δ 10.15 (s, 1H), 7.63 (d, *J* = 7.4 Hz, 1H), 7.56 (t, *J* = 7.4 Hz, 2H), 7.46 (d, *J* = 7.3 Hz, 2H), 6.68 (d, *J* = 2.0 Hz, 1H), 6.38 (dd, *J* = 8.3, 2.0 Hz, 1H), 5.26 (d, *J* = 8.3 Hz, 1H), 1.69 (s, 6H), 1.31 − 1.19 (m, 4H).^13^C NMR (126 MHz, DMSO-*d6*): δ 168.61, 168.23, 158.93, 157.08, 136.05, 131.02, 130.47, 130.30, 129.71, 128.82, 128.73, 124.84, 113.42, 113.23, 110.80, 109.36, 58.54, 57.91. ESI-HRMS(*m/z*) calculate for C_20_H_20_ClN_2_O^+^ [M + H]^+^ 339.1259 found 339.1247.

*6-chloro-3-(morpholino(phenyl)methylene)indolin-2-one (H9)*: Yellow solid; yield: 65.9%; HPLC purity: 99.44%.^1^H NMR (500 MHz, DMSO-*d6*): δ 10.23 (s, 1H), 7.64 (t, *J* = 7.3 Hz, 1H), 7.57 (t, *J* = 7.3 Hz, 2H), 7.52 − 7.49 (m, 2H), 6.69 (d, *J* = 2.0 Hz, 1H), 6.40 (dd, *J* = 8.3, 2.1 Hz, 1H), 5.27 (d, *J* = 8.3 Hz, 1H), 3.77 (s, 4H), and 3.44 (s, 4H).^13^C NMR (126 MHz, DMSO-*d6*): δ 165.72, 161.20, 138.55, 135.29, 131.60, 130.65, 129.97, 126.78, 126.51, 119.40, 118.96, 108.23, 97.47, 67.15, and 40.46. ESI-HRMS(*m/z*) calculate for C_19_H_18_ClN_2_O_2_^+^ [M + H]^+^ 341.1051 found 341.1036.

*6-chloro-3-(((3-chlorophenyl)amino)(phenyl)methylene)indolin-2-one (H10):* Yellow solid; yield: 67.3%; HPLC purity: 99.73%.^1^H NMR (500 MHz, DMSO-*d6*): δ 11.90 (s, 1H), 10.92 (s, 1H), 7.64 − 7.56 (m, 3H), 7.50 (dd, *J* = 7.8, 1.4 Hz, 2H), 7.17 (t, *J* = 8.0 Hz, 1H), 7.05 (dd, *J* = 8.0, 0.9 Hz, 1H), 6.88 (d, *J* = 2.0 Hz, 1H), 6.83 − 6.78 (m, 2H), 6.62 (dd, *J* = 8.4, 2.0 Hz, 1H), and 5.77 (d, *J* = 8.4 Hz, 1H).^13^C NMR (126 MHz, DMSO-*d6*): δ 170.53, 156.35, 140.50, 138.41, 133.56, 132.57, 130.95, 130.87, 130.04, 128.98, 128.52, 124.49, 123.17, 122.69, 121.83, 120.28, 119.60, 109.62, and 98.57. ESI-HRMS(*m/z*) calculate for C_21_H_15_Cl_2_N_2_O^+^ [M + H]^+^ 381.0556 found 381.0544.

*6-chloro-3-(phenyl(thiazol-2-ylamino)methylene)indolin-2-one (H11)*: Yellow solid; yield: 48.1%; HPLC purity: 99.88%.^1^H NMR (500 MHz, DMSO-*d6*): δ 10.19 (s, 1H), 7.62 − 7.58 (m, 1H), 7.53 (t, *J* = 7.5 Hz, 2H), 7.48 − 7.44 (m, 2H), 6.65 (d, *J* = 2.0 Hz, 1H), 6.36 (dd, *J* = 8.3, 2.0 Hz, 1H), 5.23 (d, *J* = 8.3 Hz, 1H), 3.73 (s, 4H), and 3.40 (s, 4H).^13^C NMR (126 MHz, DMSO-*d6*): δ 171.30, 167.06, 166.81, 136.19, 130.97, 130.82, 129.35, 129.09, 127.61, 125.37, 124.53, 123.42, 115.40, 105.09, 58.48, and 47.05. ESI-HRMS(*m/z*) calculate for C_18_H_13_ClN_3_O_S_^+^ [M + H]^+^ 354.0462 found 354.0452.

### Statistical analysis

All statistical analyses were performed using GraphPad Prism 8 (GraphPad Software, Inc.). Statistical analysis was carried out using a t-test or Bonferroni multiple comparisons test. A *p* values of less than 0.05 was considered significant. Unless otherwise stated, experiments were conducted in triplicate with at least three independent cultures. Multiple groups statistical analyses were determined by one-way ANOVA using GraphPad Prism 8.

## Supplementary Material

Supplemental Material
