# Supplementary material for: Searching for novel MDM2/MDMX dual inhibitors through a drug repurposing approach
Source: J Enzyme Inhib Med Chem. 2023 Dec 7;39(1):2288810. doi: 10.1080/14756366.2023.2288810 (PMC11721856; doi:10.1080/14756366.2023.2288810)
Supplement: Supplemental Material [file IENZ_A_2288810_SM5233.pdf]

Supplementary information

Searching for novel MDM2/MDMX dual inhibitors through a drug repurposing approach

Keting Li [1], Wenshu Hu[1], Yingjie Wang, Wenxing Chen, Hongmei Wen, Jian Liu, Wei Li [*], Bo Wang [*]

School of Pharmacy, Nanjing University of Chinese Medicine, Nanjing 210023, China

[*]Corresponding authors, E-mail: liwaii@njucm.edu.cn (W. Li), bwang@njucm.edu.cn (B. Wang)

[1] These authors contributed equally to this work.

1. Molecular Redocking of RO2334 with MDM2/MDMX and the residue contribution study............- 2 -
2. Molecular docking of nintedanib with MDM2/MDMX and the residue contribution study............- 4 -
3. Hydrogen bonds formation and occupancy study ....- 6 -
4. NMR and HPLC spectra of the corresponding compounds ....- 7 -

1. Molecular Redocking of RO2334 with MDM2/MDMX and the residue contribution study

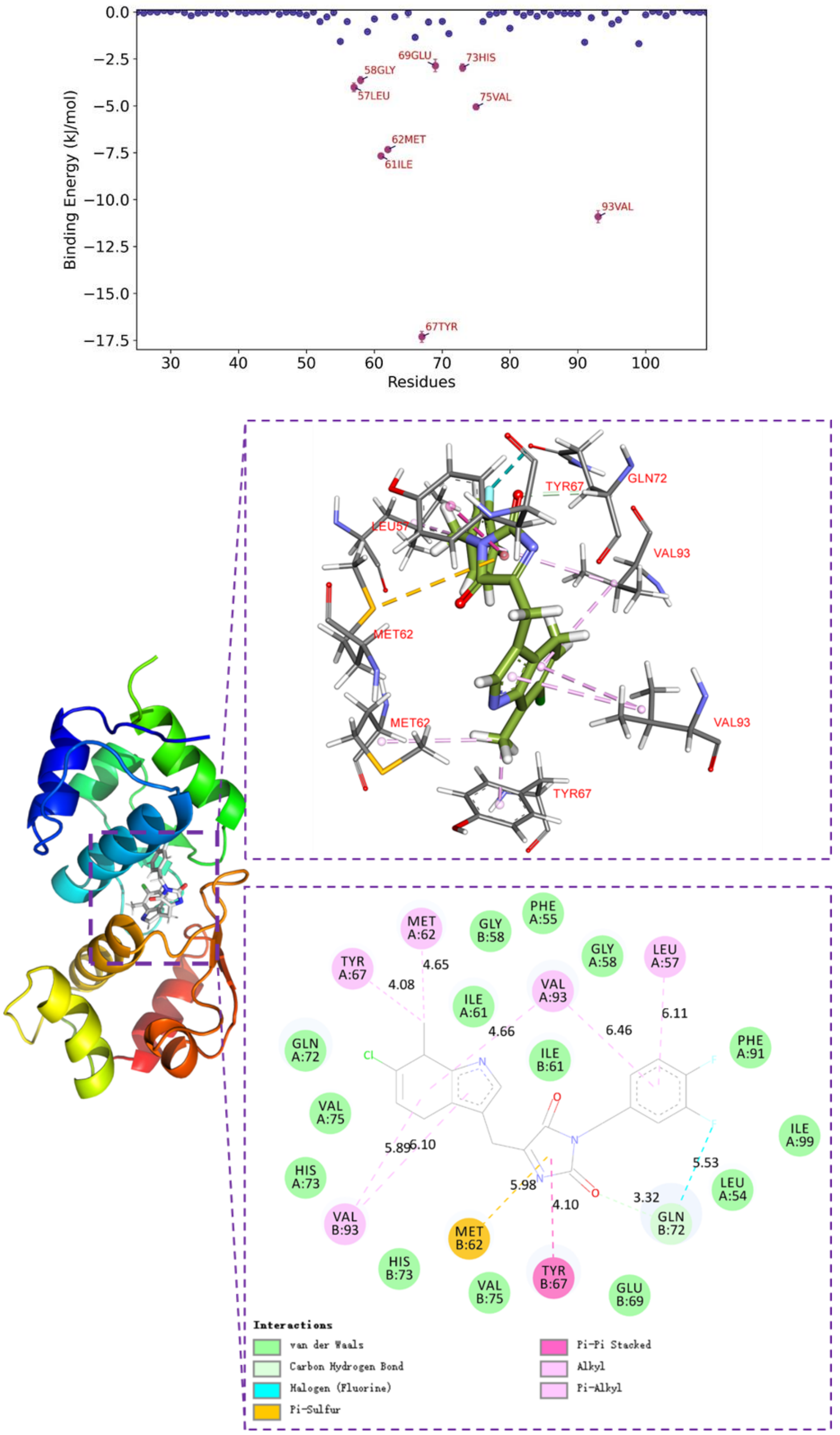

Figure S1. Residue contribution and protein-ligand interaction diagram of MDM2- RO2433

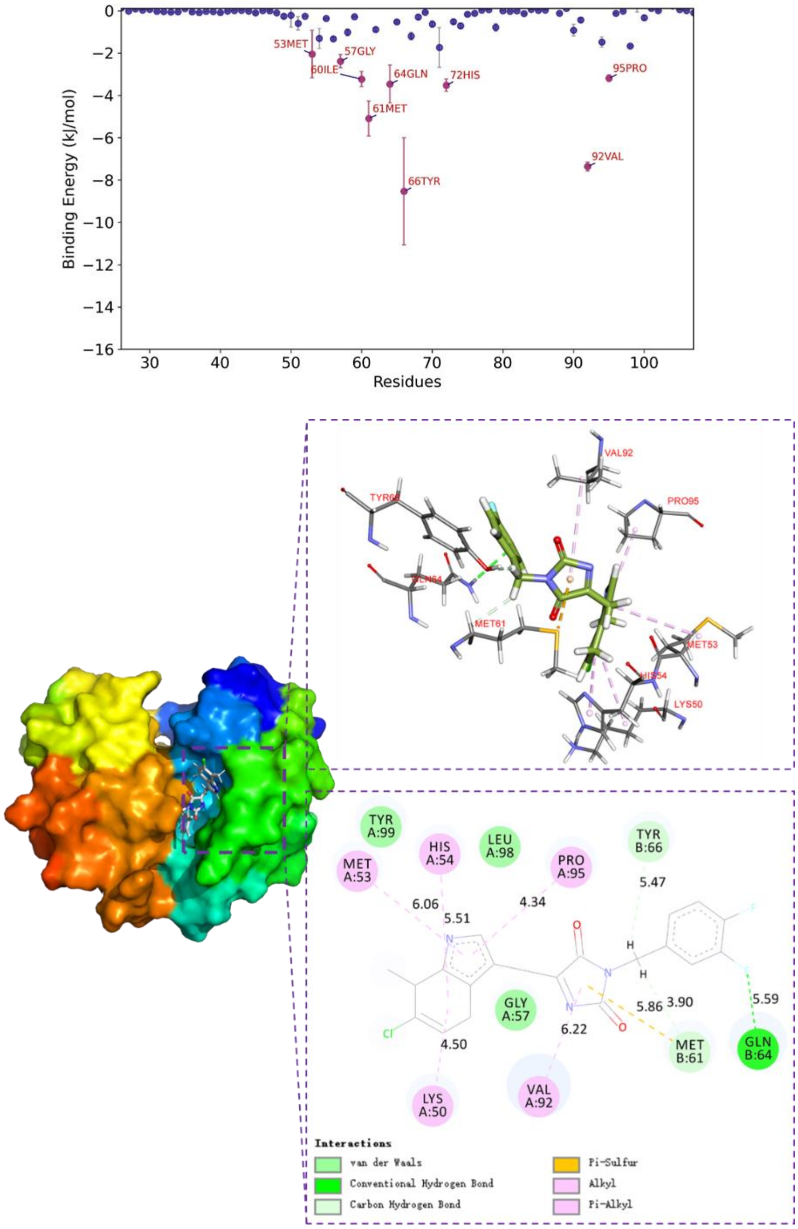

Figure S2. Residue contribution and protein-ligand interaction diagram of MDMX- RO2433

2. Molecular docking of nintedanib with MDM2/MDMX and the residue contribution study

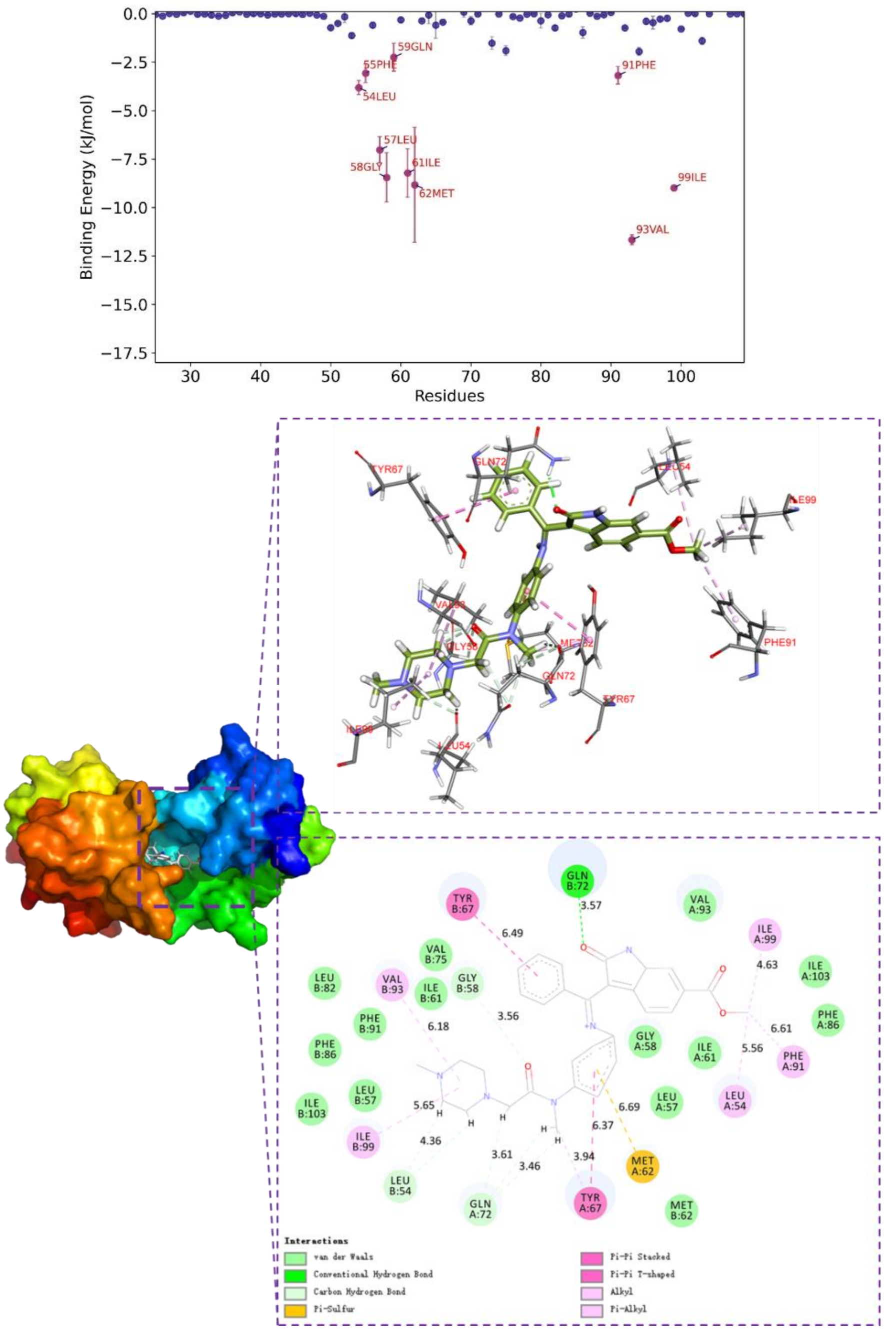

Figure S3. Residue contribution and protein-ligand interaction diagram of MDM2-Nintedanib

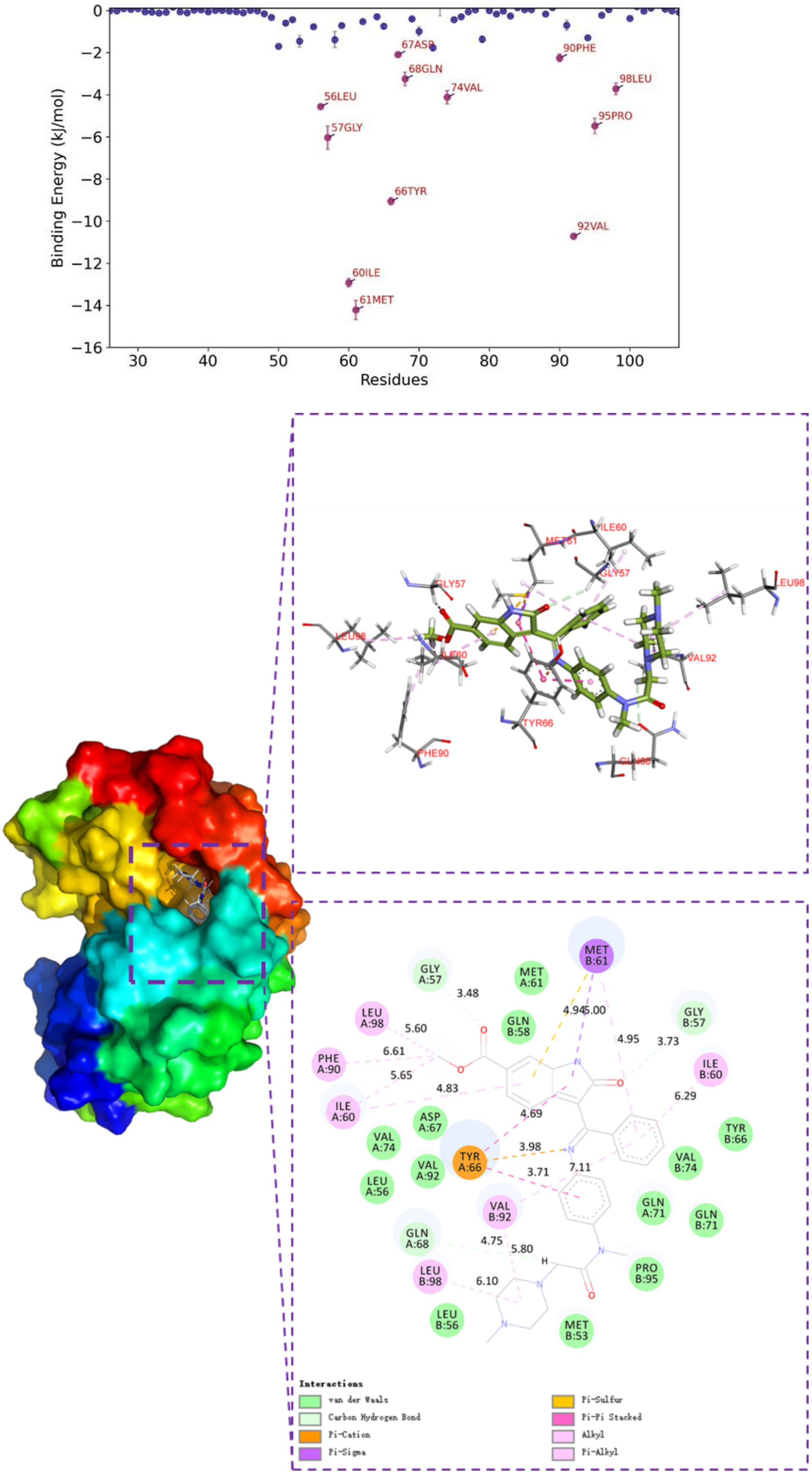

Figure S4. Residue contribution and protein-ligand interaction diagram of MDMX-Nintedanib

3. Hydrogen bonds formation and occupancy study

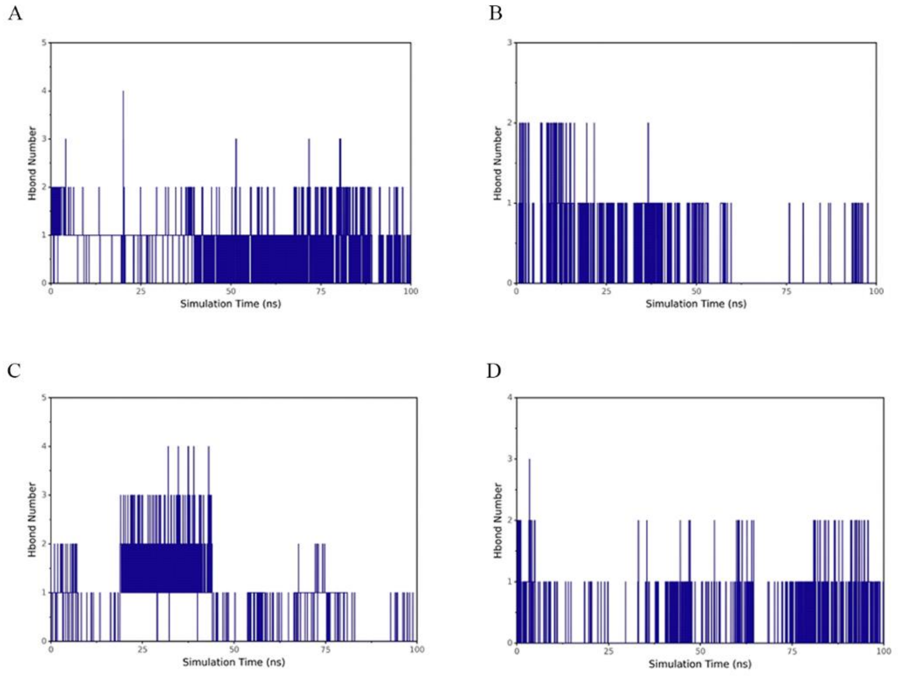

Figure S5. Hydrogen bonds formed in different complexes. (A) MDM2-nintedanib complex; (B) MDM2-RO2433 complex; (C) MDMX-nintedanib complex; (D) MDMX-RO2433 complex.

A

Index	Donor	Acceptor	Occupancy%
1	65ARG(chain_A#NH1)	Ligand(2911#O36)	2.8%
2	67TYR(chain_A#OH)	Ligand(2876#N1)	19.0%
3	67TYR(chain_A#OH)	Ligand(2911#O36)	1.3%
4	67TYR(chain_B#OH)	Ligand(2889#O14)	41.6%
5	72GLN(chain_B#NE2)	Ligand(2889#O14)	2.5%
6	Ligand(2876#N1)	67TYR(chain_B#OH)	14.2%

B

Index	Donor	Acceptor	Occupancy%
1	72GLN(chain_B#NE2)	Ligand(2881#N)	4.0%
2	72GLN(chain_B#NE2)	Ligand(2888#N)	1.4%
3	72GLN(chain_B#NE2)	Ligand(2892#O)	18.0%

C

Index	Donor	Acceptor	Occupancy%
1	68GLN(chain_A#NE2)	Ligand(2708#O36)	2.3%
2	99TYR(chain_B#N)	Ligand(2686#O14)	10.5%
3	Ligand(2673#N1)	71GLN(chain_A#OE1)	7.1%
4	Ligand(2683#N11)	58GLN(chain_B#OE1)	43.3%

D

Index	Donor	Acceptor	Occupancy%
1	71GLN(chain_A#NE2)	Ligand(2689#O)	1.9%
2	64GLN(chain_B#NE2)	Ligand(2685#N)	4.8%
3	64GLN(chain_B#NE2)	Ligand(2689#O)	1.4%
4	66TYR(chain_B#N)	Ligand(2689#O)	1.2%
5	71GLN(chain_B#NE2)	Ligand(2689#O)	2.5%

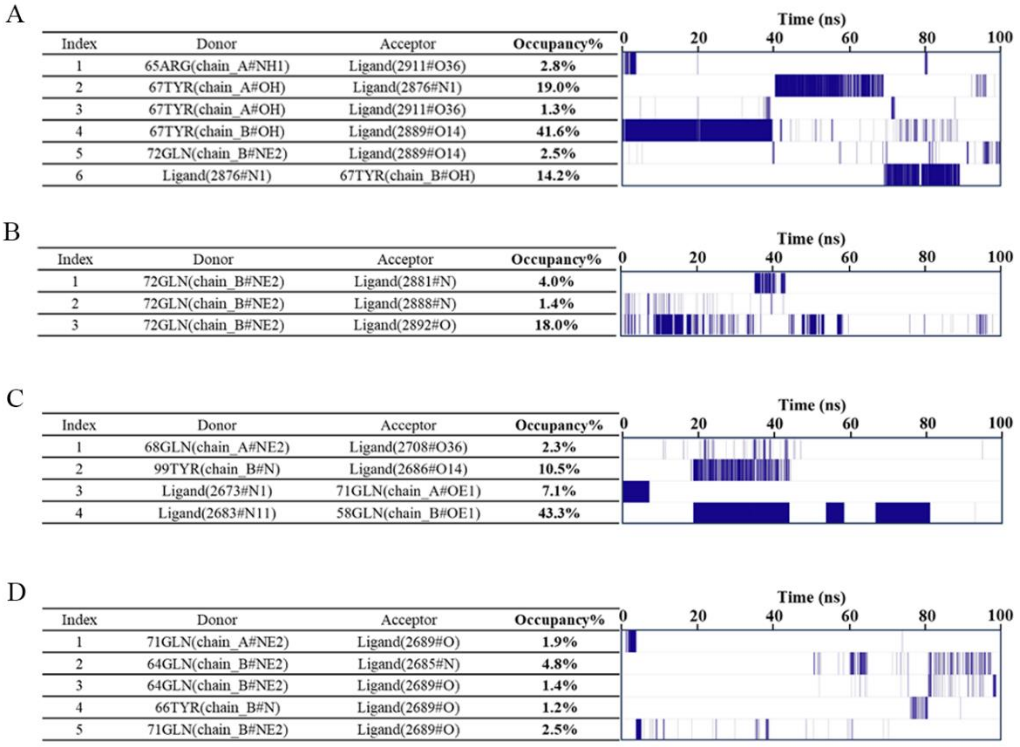

Figure S6. Hydrogen bonds occupancy analysis. (A) MDM2-nintedanib complex; (B) MDM2-RO2433 complex; (C) MDMX-nintedanib complex; (D) MDMX-RO2433 complex.

4. NMR and HPLC spectra of the corresponding compounds

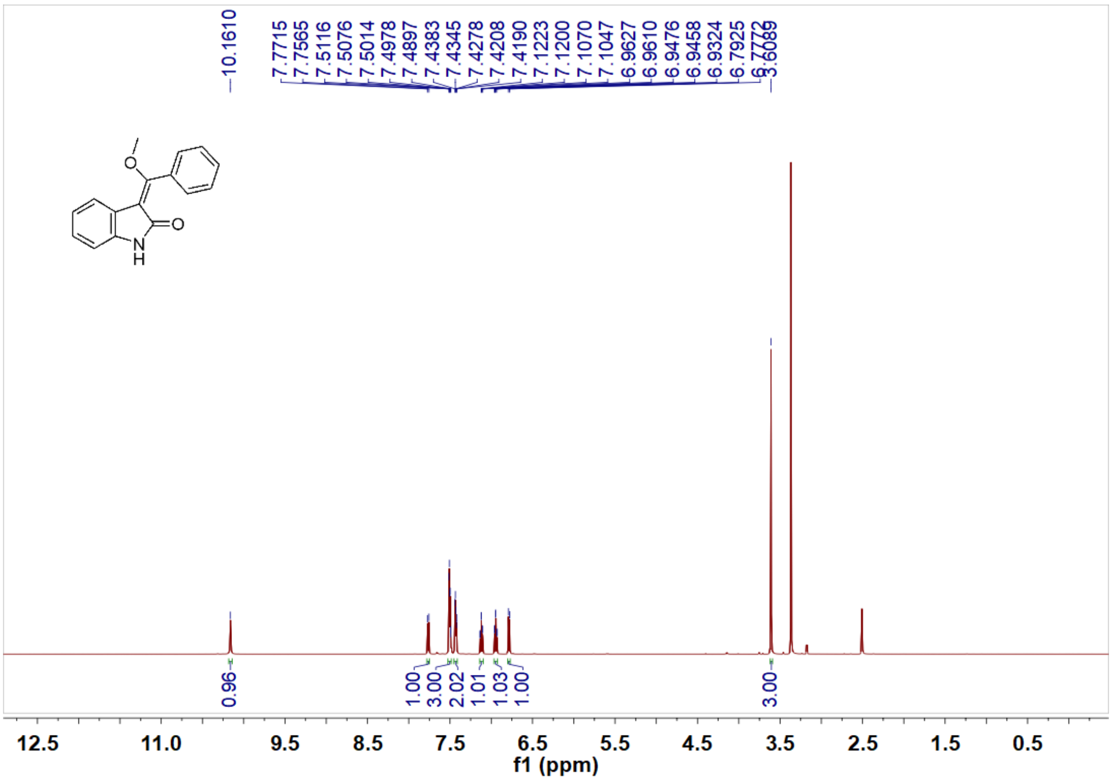

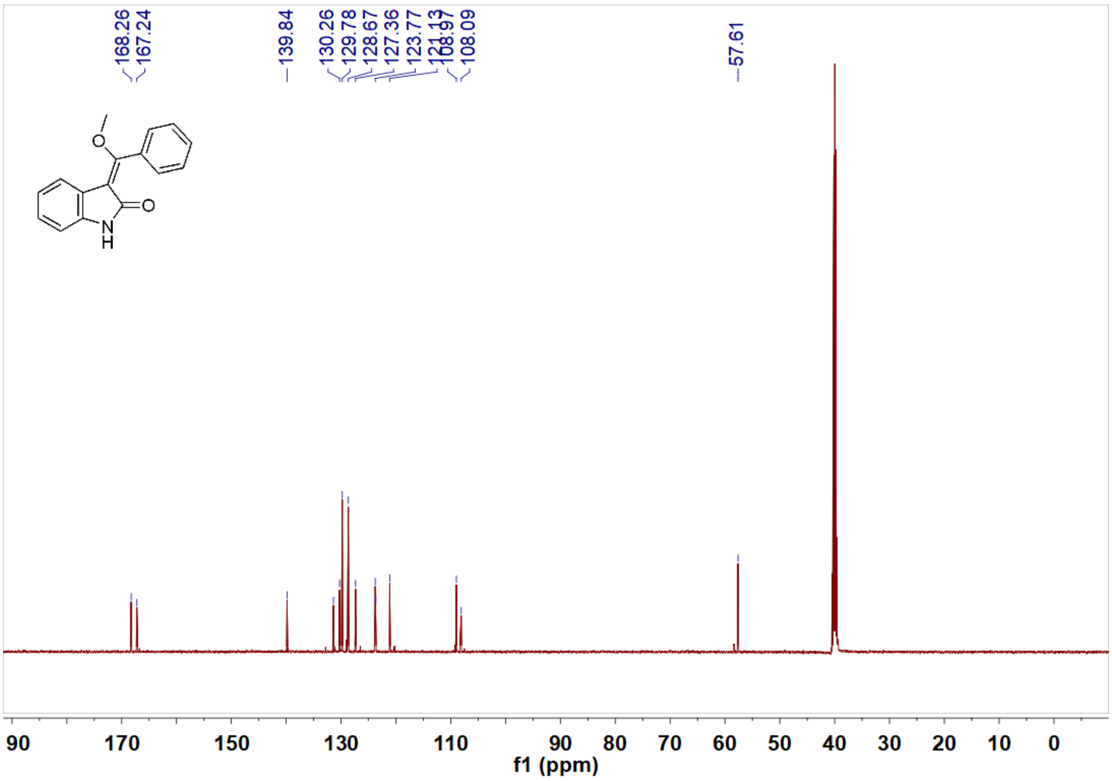

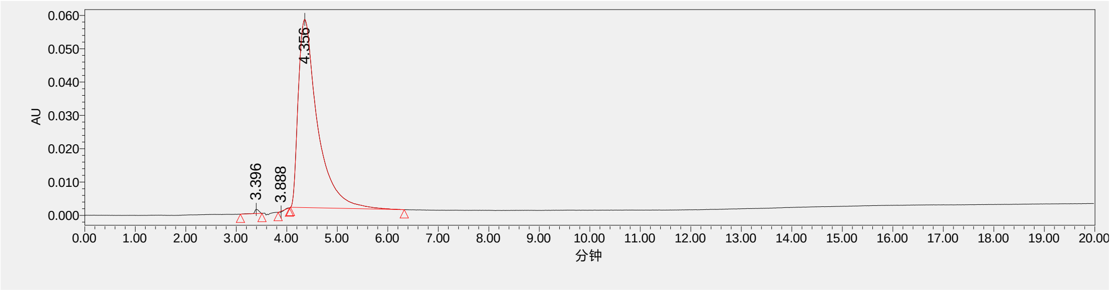

Peak	RetTime (min)	Area (mAU*s)	Area (%)
1	3.396	7548	0.50
2	3.888	1834	0.12
3	4.356	1493318	99.38

Figure S7. NMR and HPLC spectra of compound H1

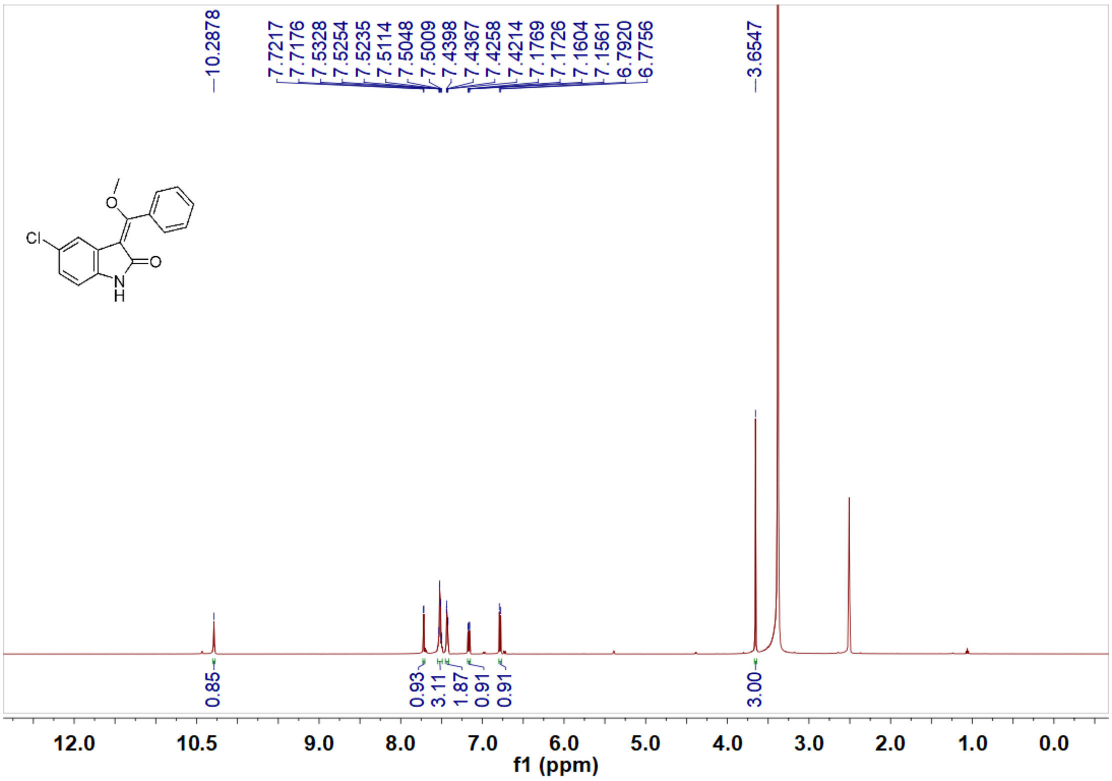

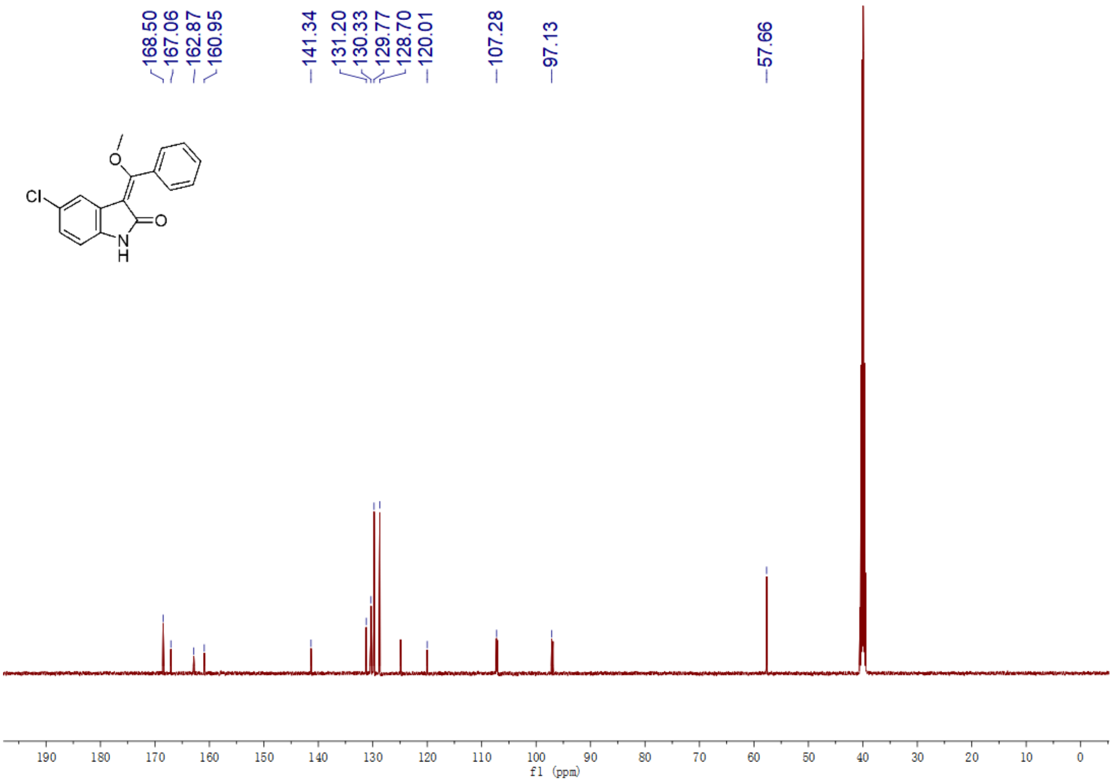

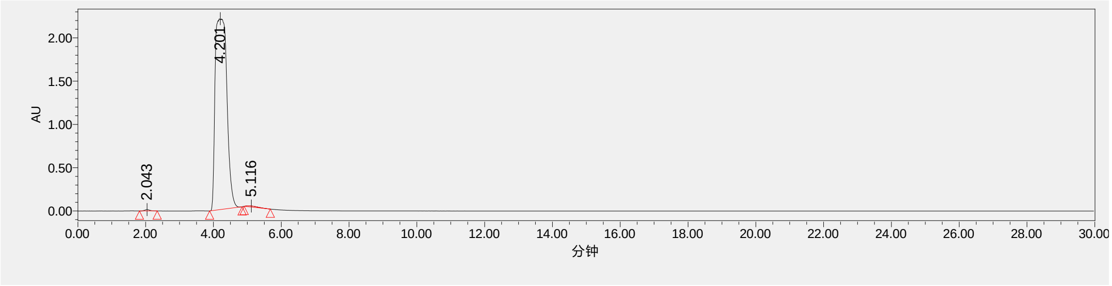

Peak	RetTime (min)	Area (mAU*s)	Area (%)
1	2.043	164188	0.31
2	4.201	53012887	99.15
3	5.116	288424	0.54

Figure S8. NMR and HPLC spectra of compound H2

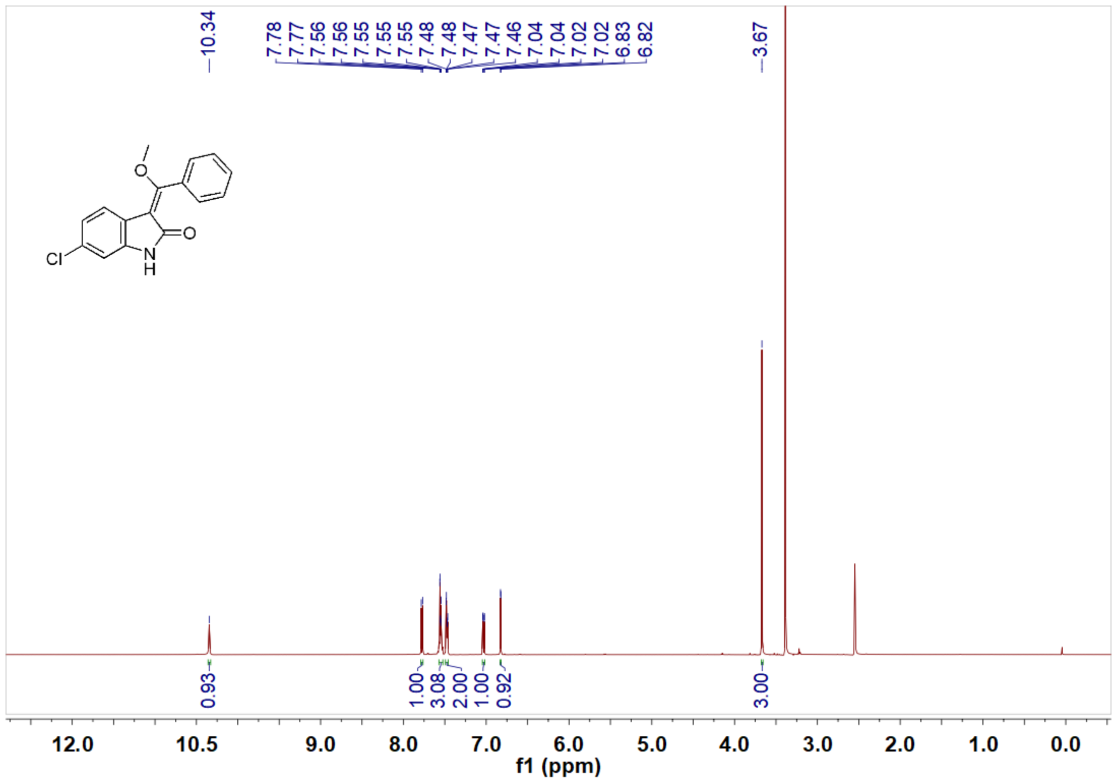

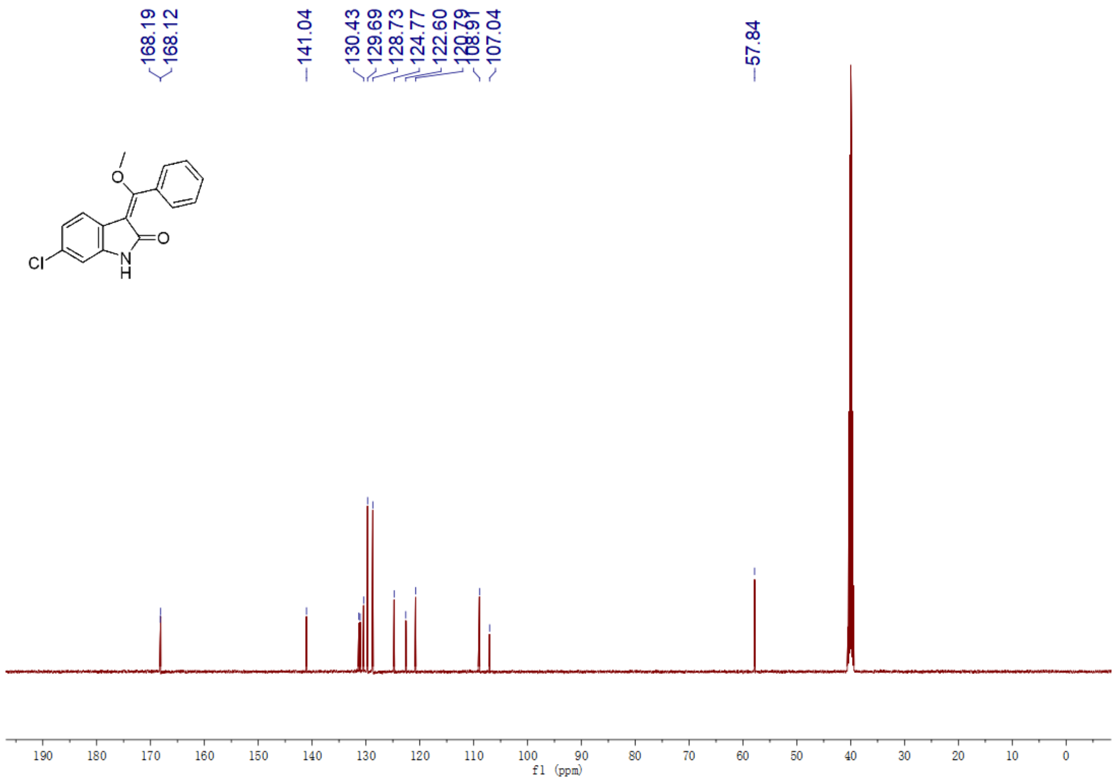

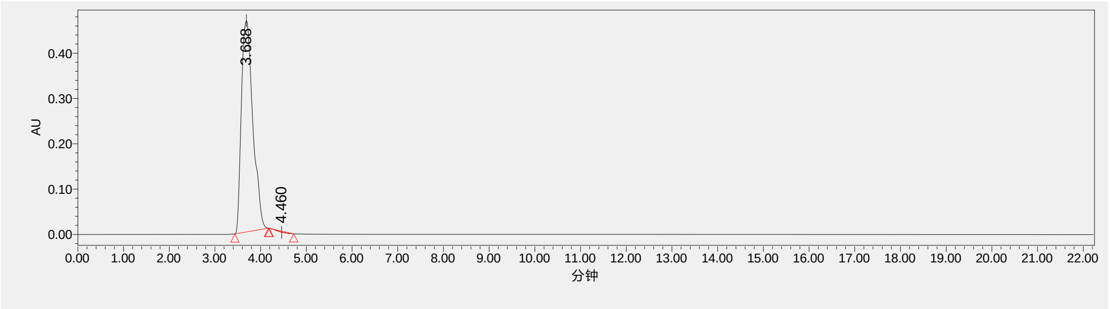

Peak	RetTime (min)	Area (mAU*s)	Area (%)
1	3.688	8131285	99.47
2	4.460	43195	0.53

Figure S9. NMR and HPLCspectra of compound H3

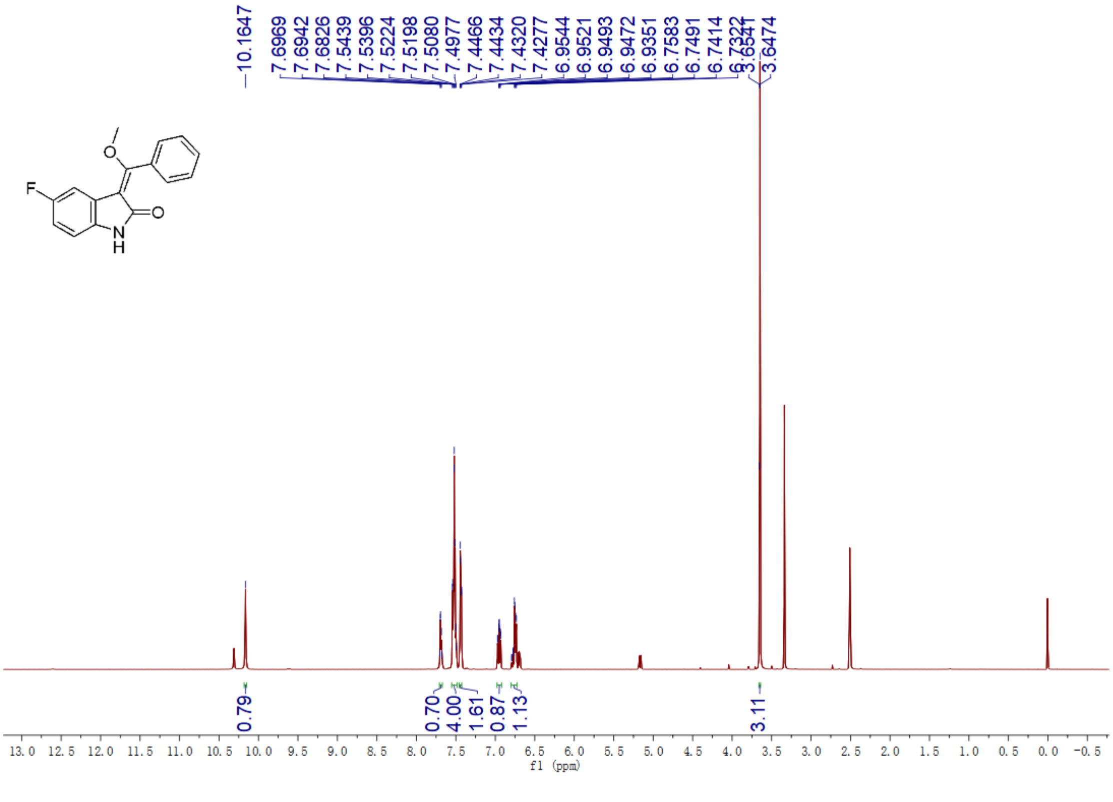

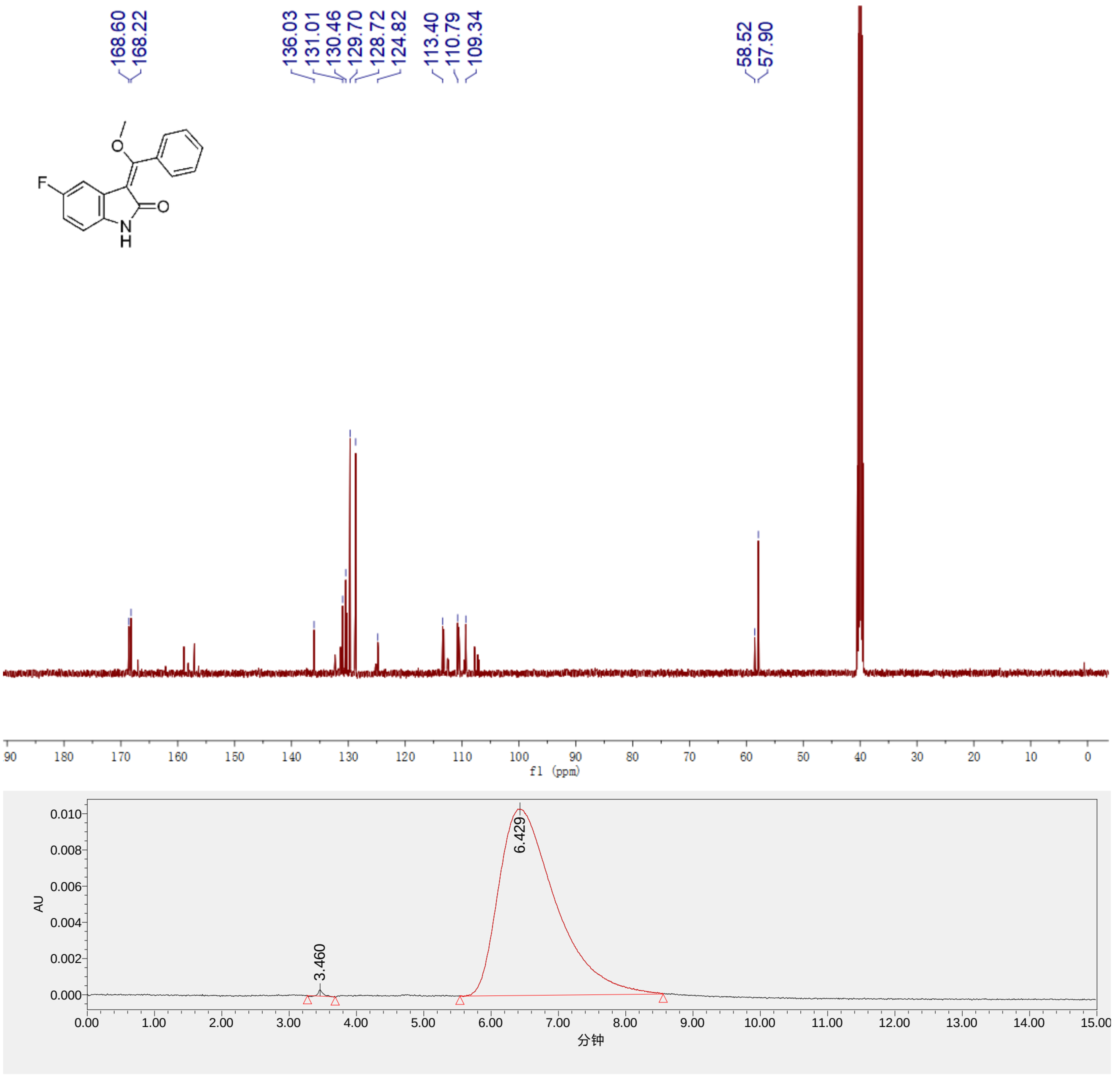

Peak	RetTime (min)	Area (mAU*s)	Area (%)
1	3.460	1953	0.32
2	6.429	605537	99.68

Figure S10. NMR and HPLC spectra of compound H4

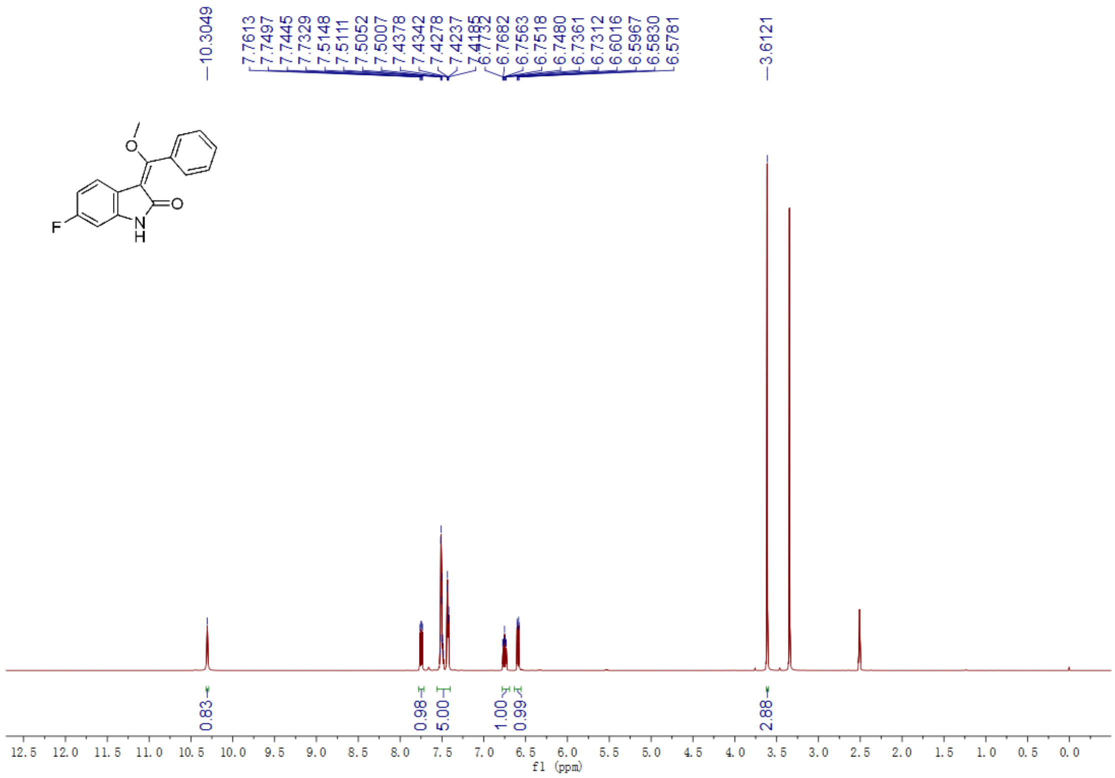
10.3049
7.7613
7.7497
7.7445
7.7329
7.5148
7.5111
7.5052
7.5007
7.4378
7.4342
7.4278
7.4237
7.4185
6.7732
6.7682
6.7563
6.7518
6.7480
6.7361
6.7312
6.6016
6.5967
6.5830
6.5781
3.6121
O
F
N
H
O
0.83
0.98
5.00
1.00
0.99
2.88
12.5 12.0 11.5 11.0 10.5 10.0 9.5 9.0 8.5 8.0 7.5 7.0 6.5 6.0 5.5 5.0 4.5 4.0 3.5 3.0 2.5 2.0 1.5 1.0 0.5 0.0
f1 (ppm)

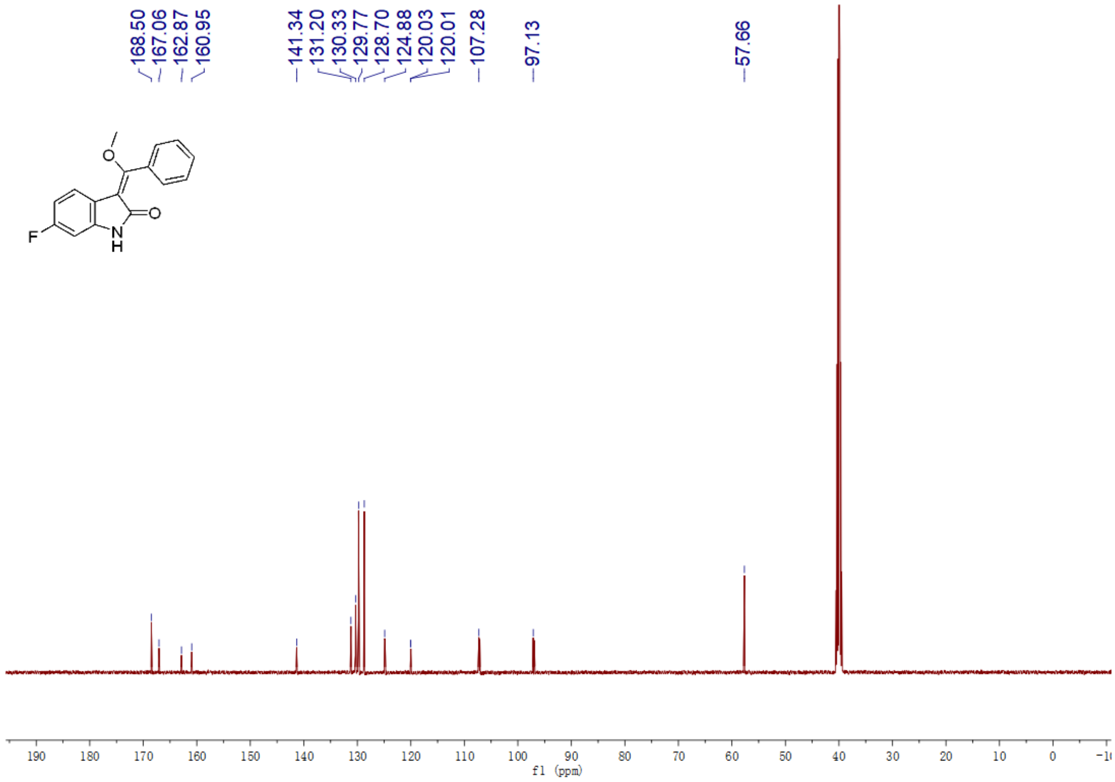
168.50
167.06
162.87
160.95
141.34
131.20
130.33
129.77
128.70
124.88
120.03
120.01
107.28
97.13
57.66
O
F
N
H
O
190 180 170 160 150 140 130 120 110 100 90 80 70 60 50 40 30 20 10 0
f1 (ppm)

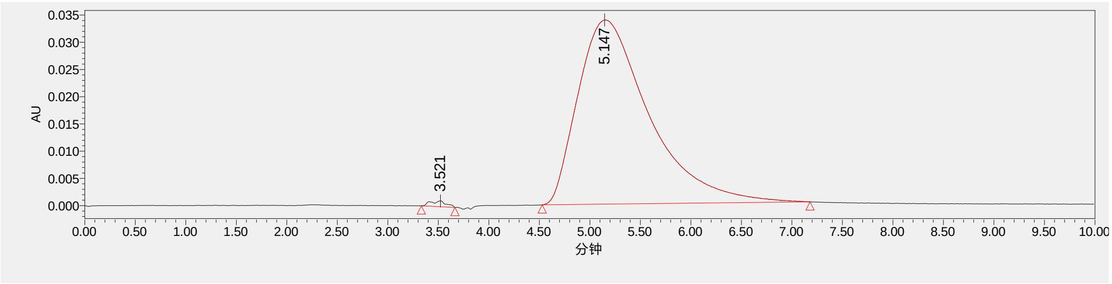

Peak	RetTime (min)	Area (mAU*s)	Area (%)
1	3.521	11397	0.68
2	5.147	1654568	99.32

Figure S11. NMR and HPLCspectra of compound H5

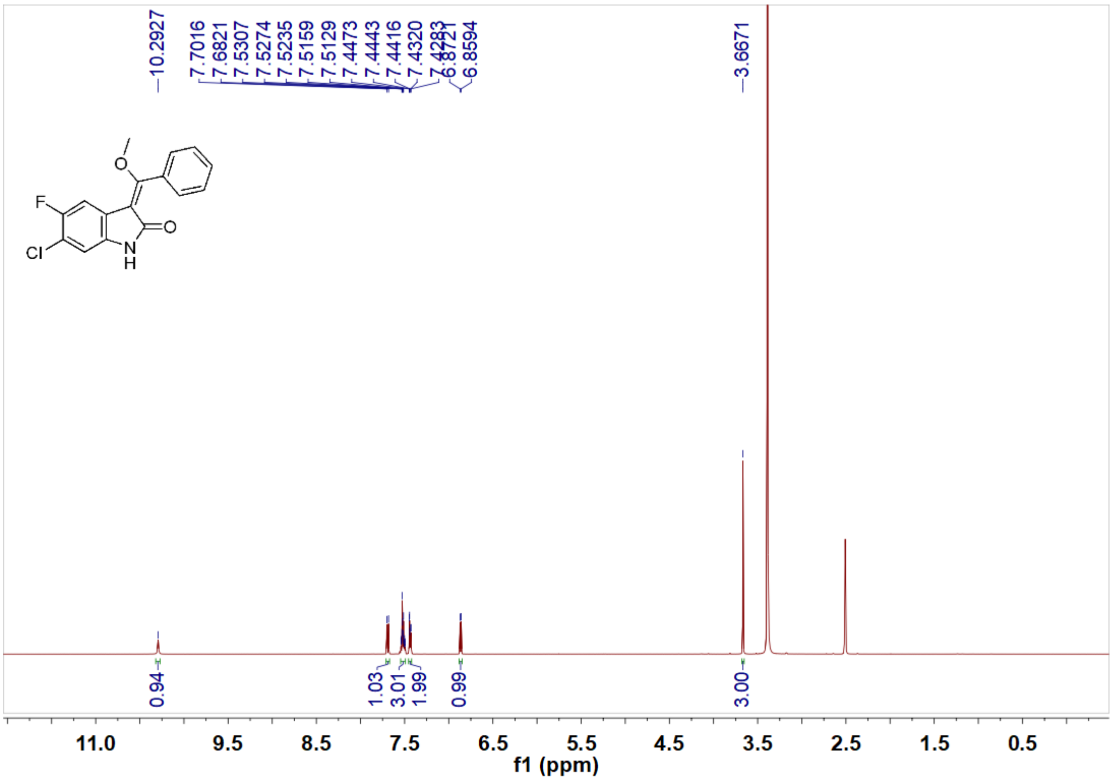

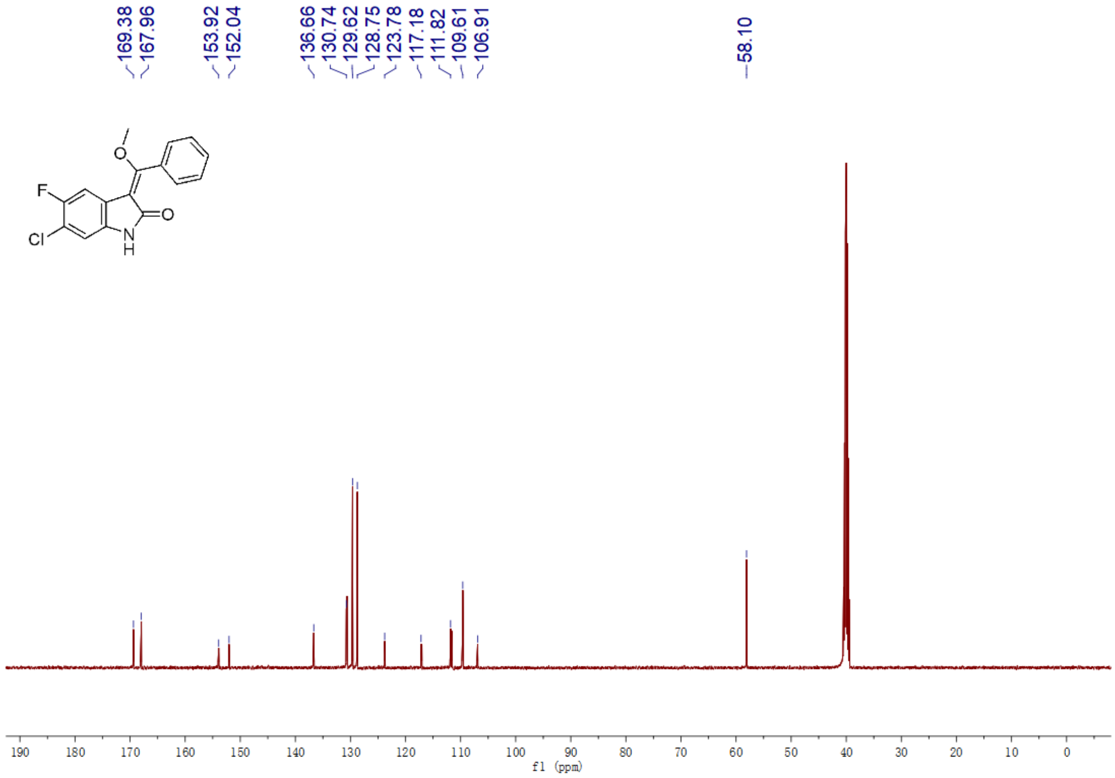

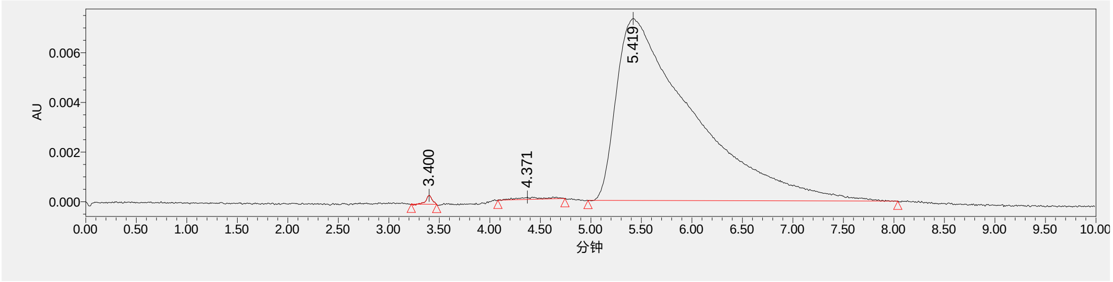

Peak	RetTime (min)	Area (mAU*s)	Area (%)
1	3.400	1591	0.41
2	4.371	1666	0.43
3	5.419	388132	99.17

Figure S12. NMR and HPLC spectra of compound H6

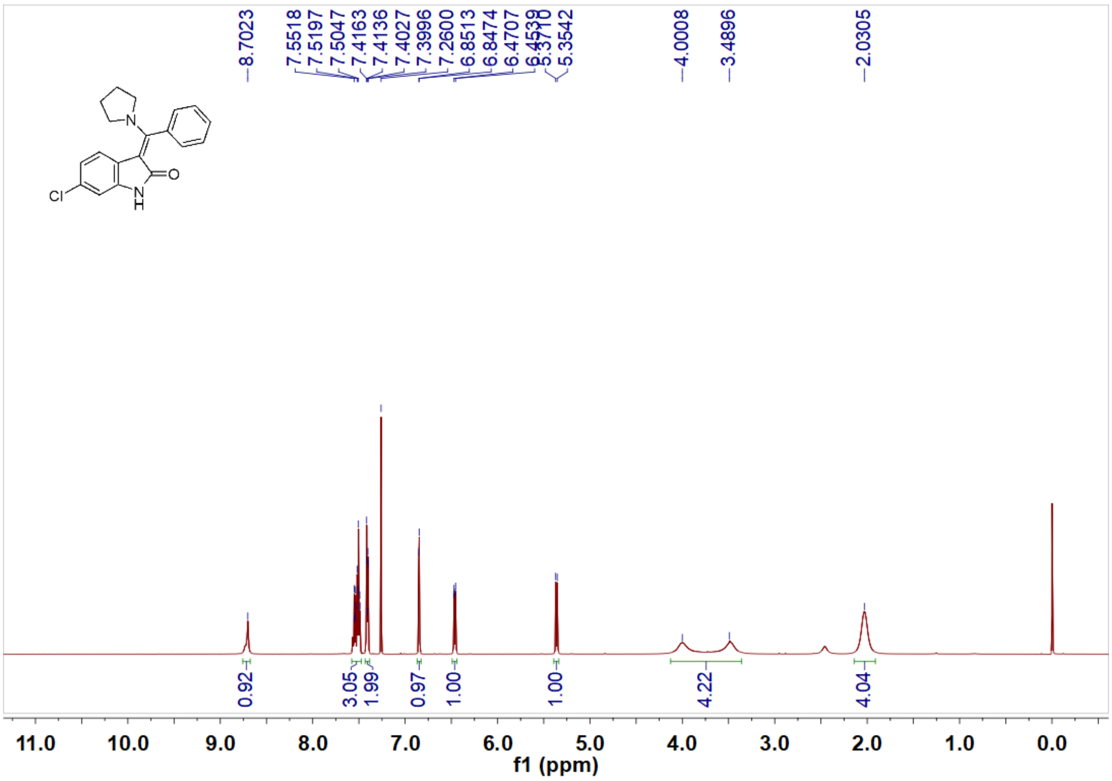
8.7023
7.5518
7.5197
7.5047
7.4163
7.4136
7.4027
7.3996
7.2600
6.8513
6.8474
6.4707
6.4539
5.3710
5.3542
4.0008
3.4896
2.0305
0.92
3.05
1.99
0.97
1.00
1.00
4.22
4.04
11.0
10.0
9.0
8.0
7.0
6.0
5.0
4.0
3.0
2.0
1.0
0.0
f1 (ppm)
Cl
N
H
O
N

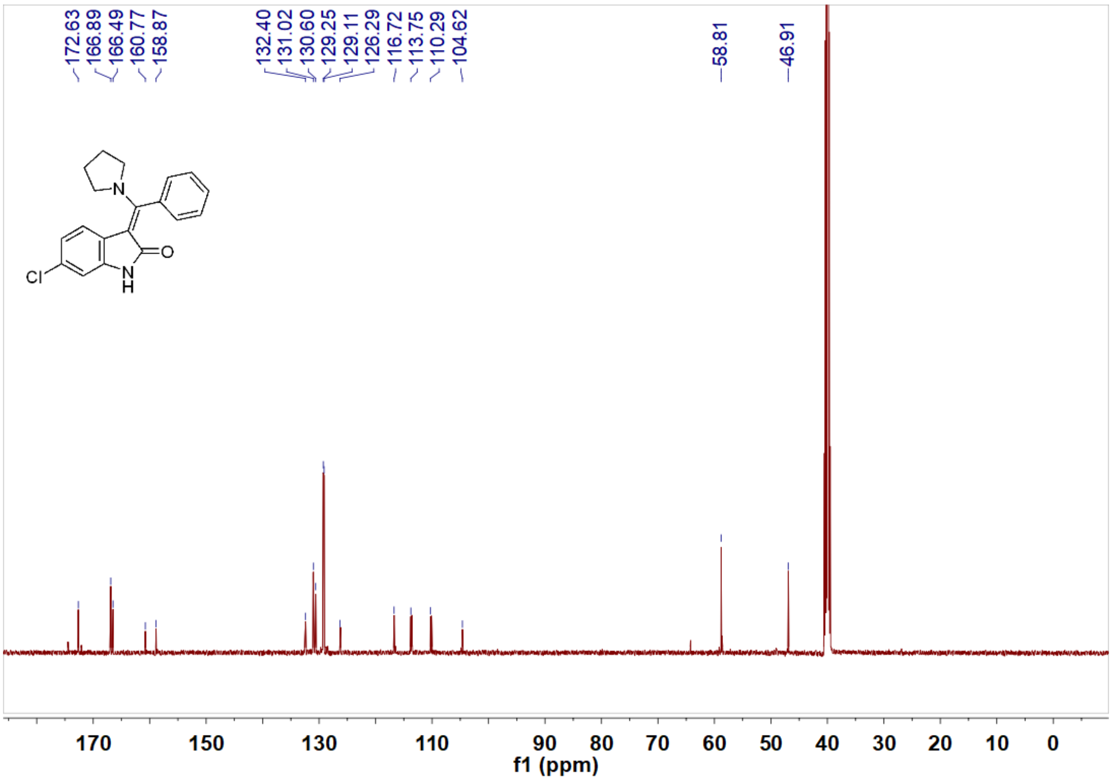
172.63
166.89
166.49
160.77
158.87
132.40
131.02
130.60
129.25
129.11
126.29
116.72
113.75
110.29
104.62
58.81
46.91
170
150
130
110
90
80
70
60
50
40
30
20
10
0
f1 (ppm)
Cl
N
H
O
N

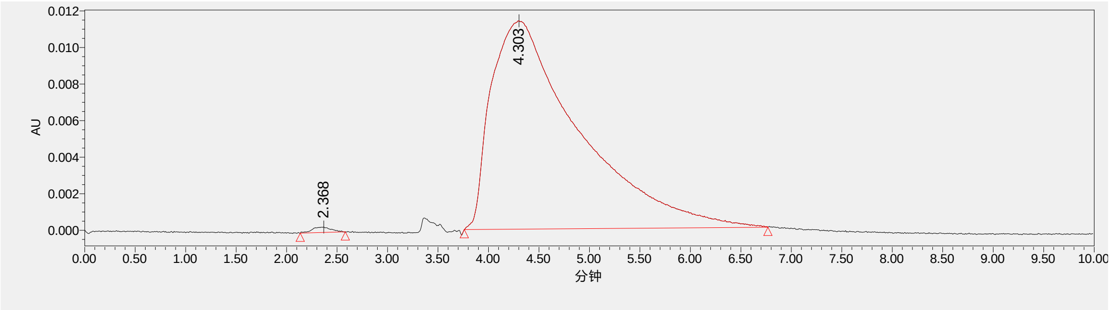

Peak	RetTime (min)	Area (mAU*s)	Area (%)
1	4.303	692247	99.44
2	2.368	3907	0.56

Figure S13. NMR and HPLC spectra of compound H7

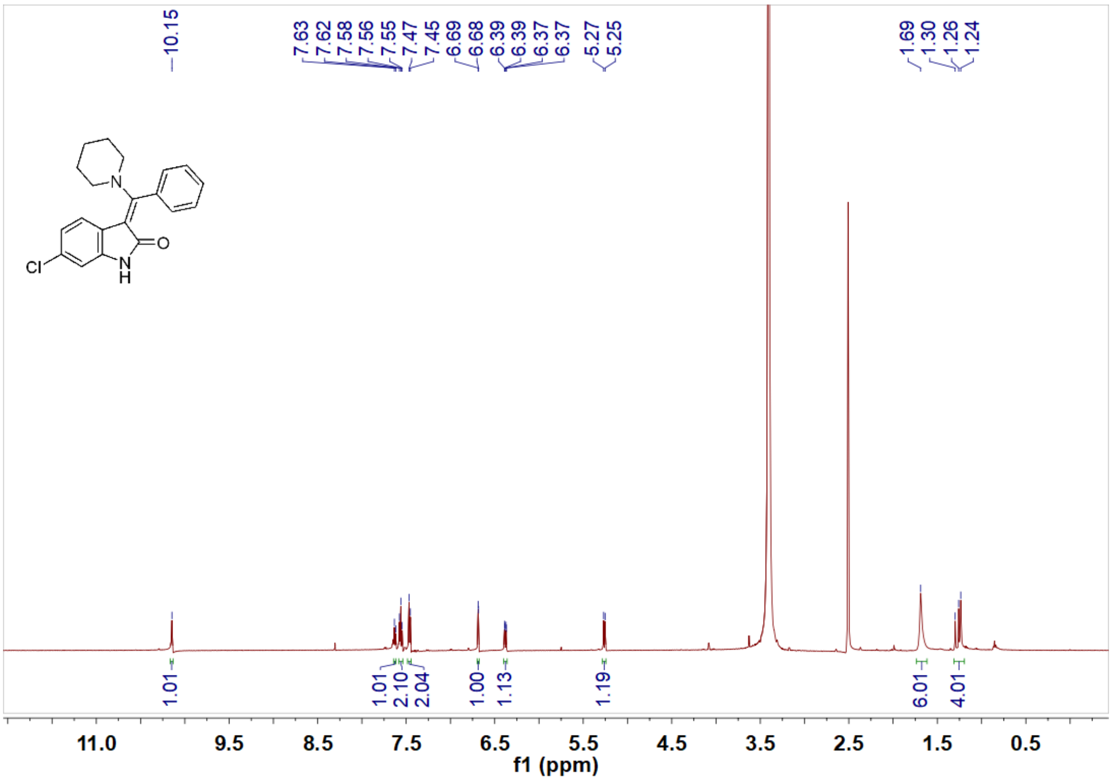

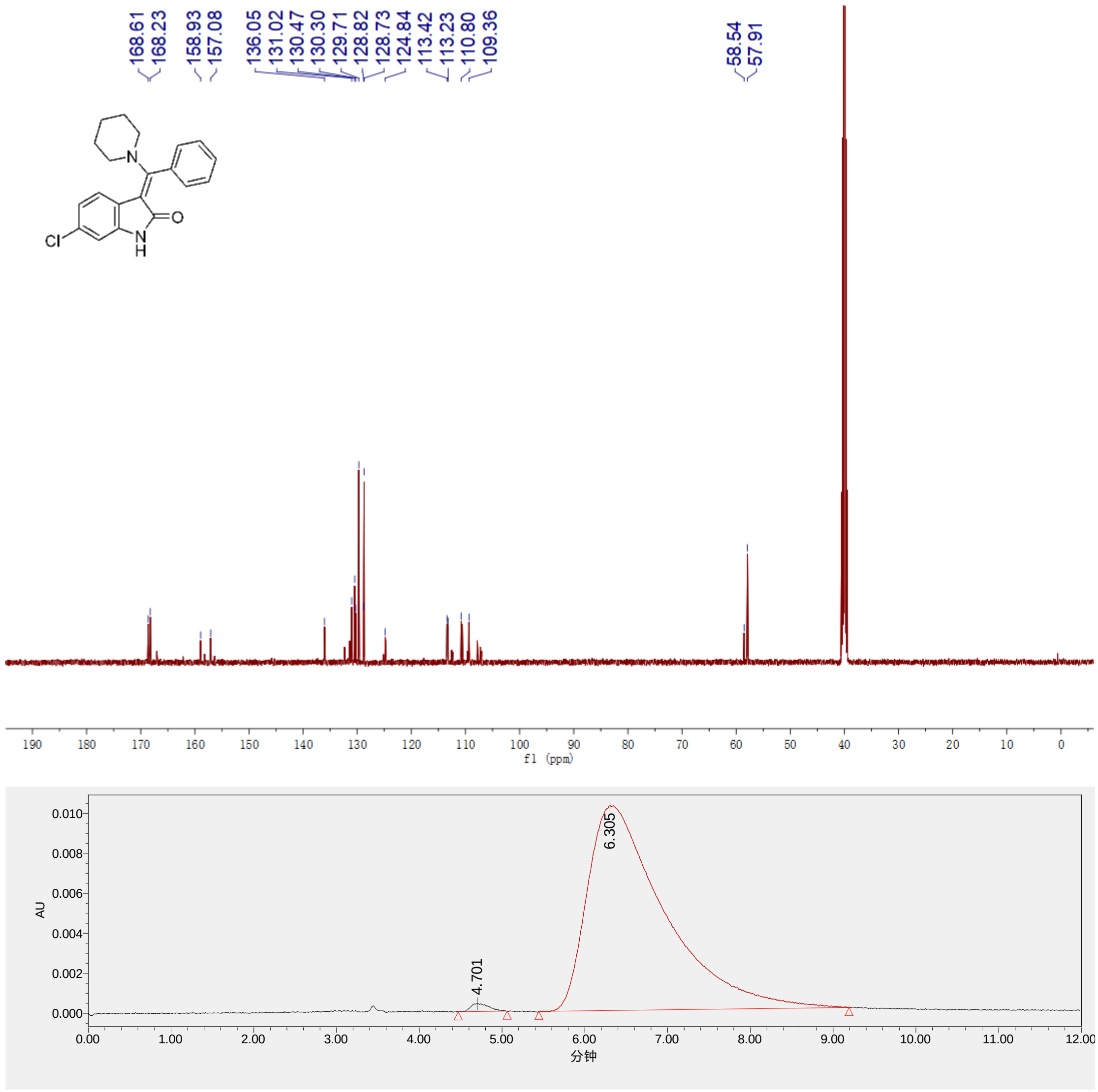

Peak	RetTime (min)	Area (mAU*s)	Area (%)
1	6.305	664649	99.09
2	4.701	6103	0.91

Figure S14. NMR and HPLC spectra of compound H8

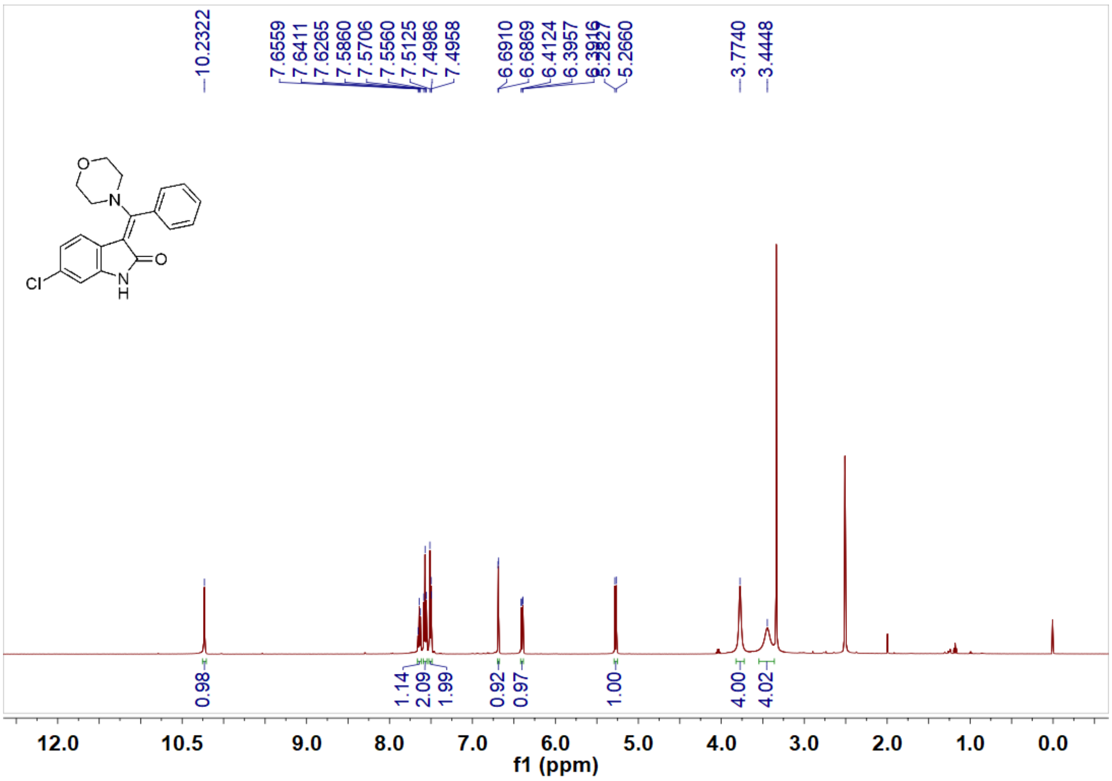

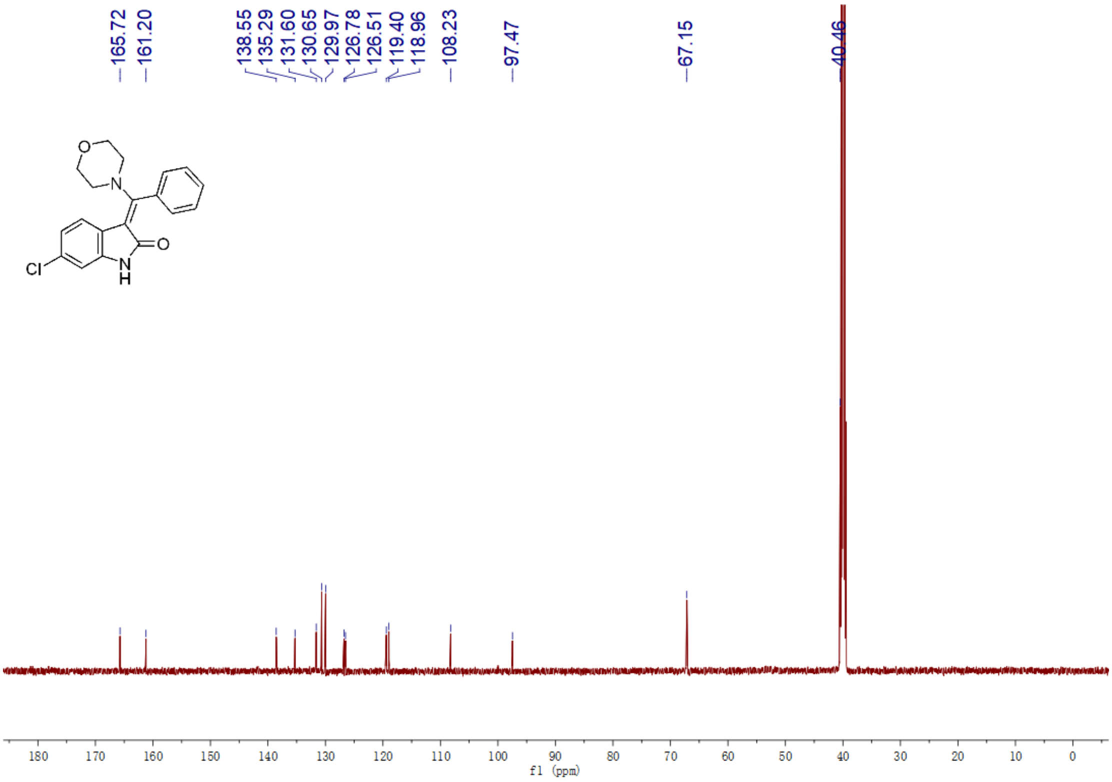

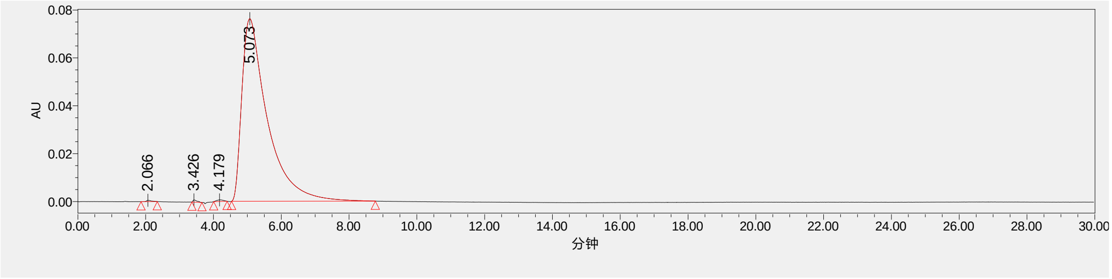

Peak	RetTime (min)	Area (mAU*s)	Area (%)
1	2.066	5615	0.14
2	3.426	7376	0.18
3	4.179	9226	0.23
4	5.073	3971230	99.44

Figure S15. NMR and HPLC spectra of compound H9

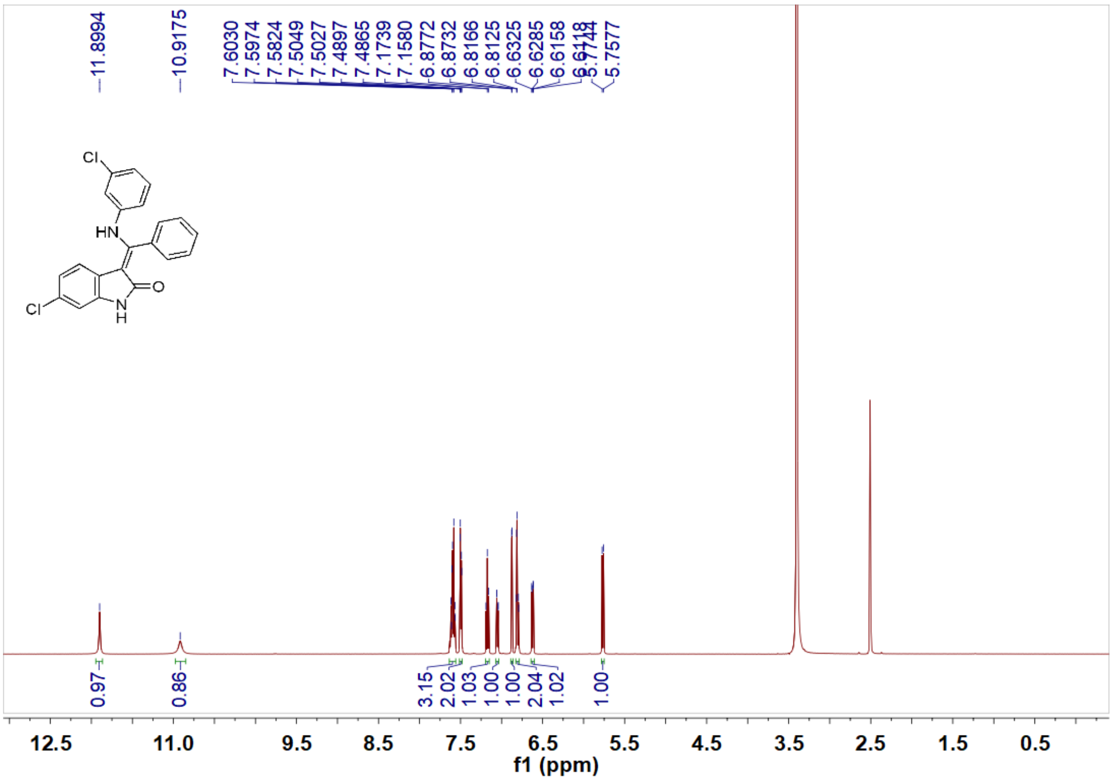

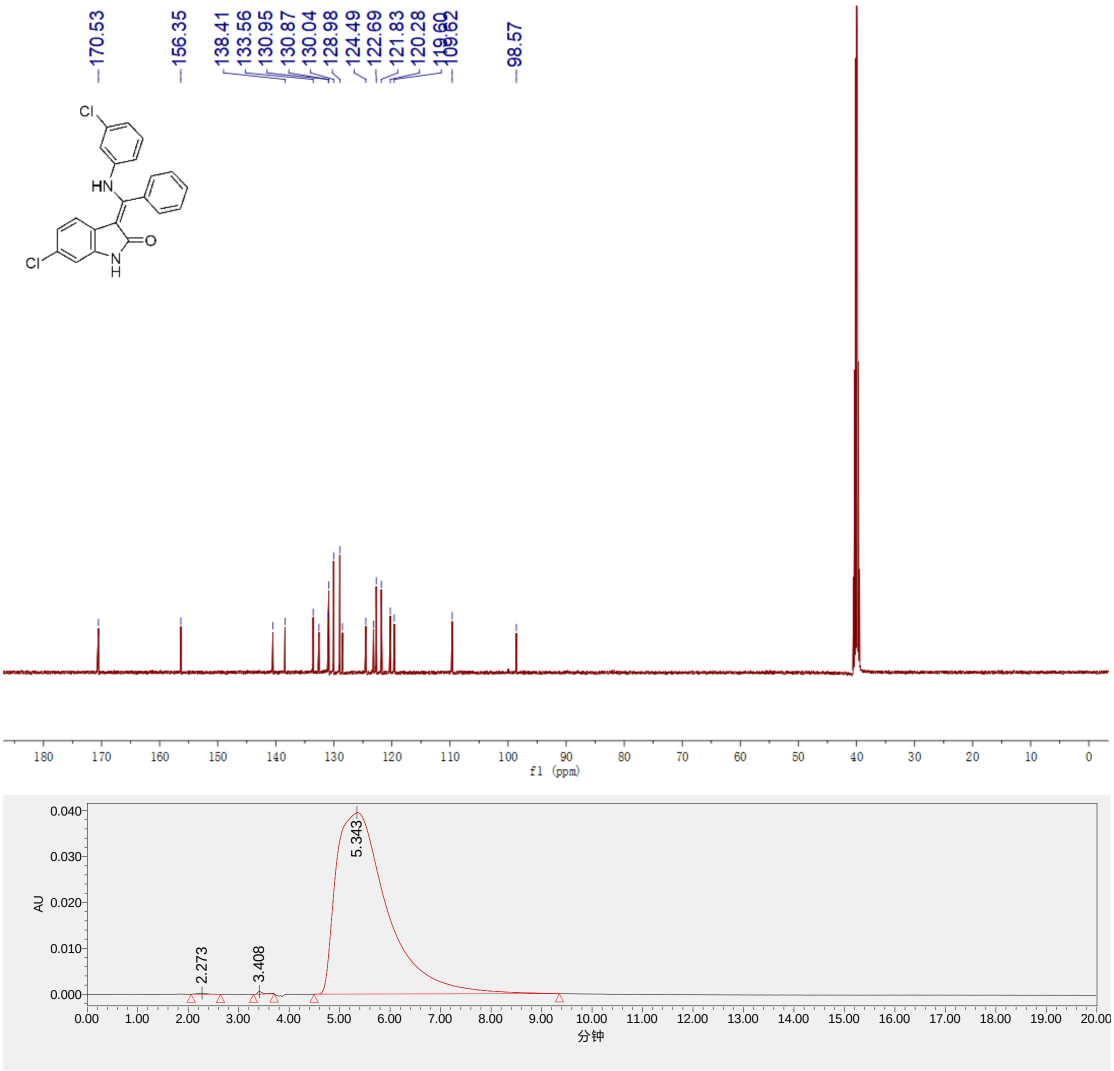

Peak	RetTime (min)	Area (mAU*s)	Area (%)
1	2.273	2903	0.10
2	3.408	4578	0.16
3	5.343	2789078	99.73

Figure S16. NMR and HPLC spectra of compound H10

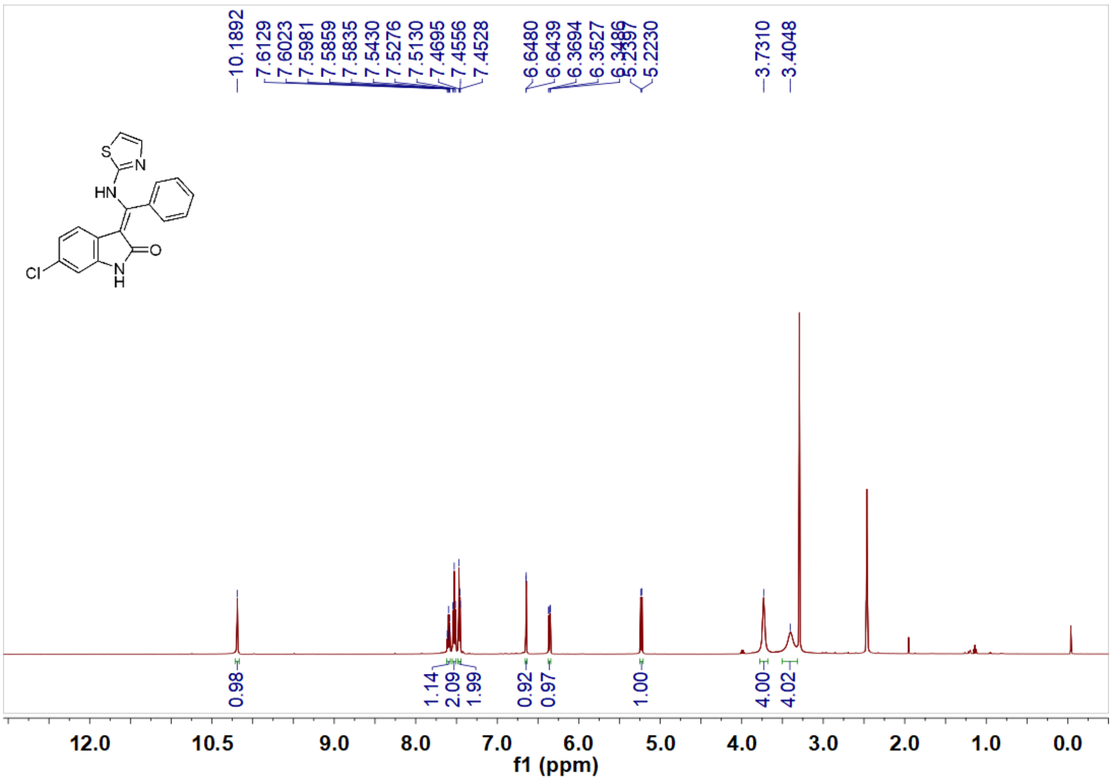
S
N
HN
O
Cl
N
H
10.1892
7.6129
7.6023
7.5981
7.5859
7.5835
7.5430
7.5276
7.5130
7.4695
7.4556
7.4528
6.6480
6.6439
6.3694
6.3527
5.2397
5.2230
3.7310
3.4048
0.98
1.14
2.09
1.99
0.92
0.97
1.00
4.00
4.02
f1 (ppm)

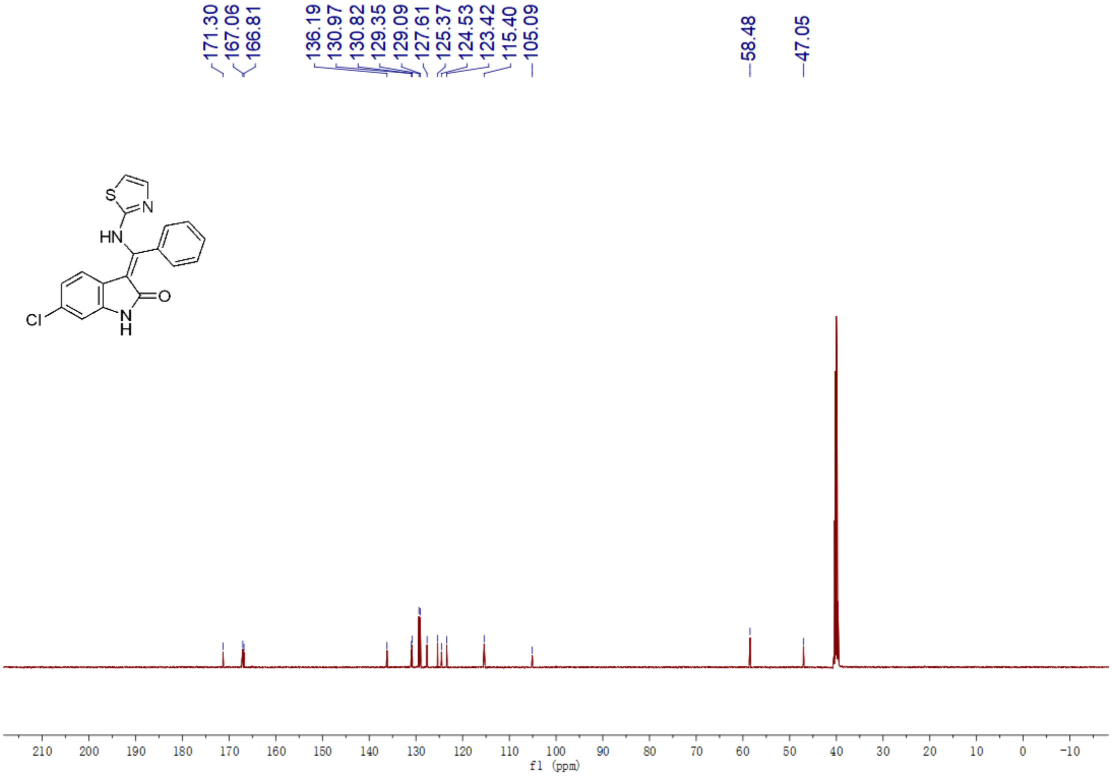
171.30
167.06
166.81
136.19
130.97
130.82
129.35
129.09
127.61
125.37
124.53
123.42
115.40
105.09
58.48
47.05
S
N
HN
O
Cl
N
H
f1 (ppm)

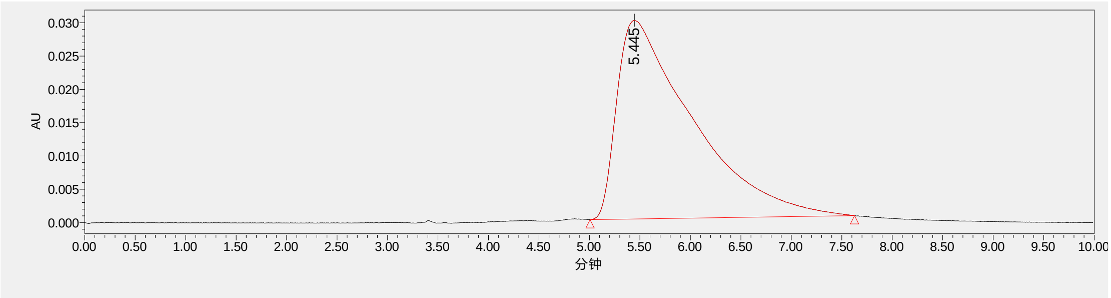

Peak	RetTime (min)	Area (mAU*s)	Area (%)
1	4.851	1729	0.12
2	5.445	1501587	99.88

Figure S17. NMR and HPLC spectra of compound H11
